# (TD)DFT investigation of donor influence on the electronic and photovoltaic properties of D-*π*-A-*π*-A quinoxaline dyes

**DOI:** 10.1007/s00894-026-06944-9

**Published:** 2026-09-16

**Authors:** Malúcia M. Soeiro, Nathália M. P. Rosa, Itamar Borges

**Affiliations:** 1 Departamento de Química, Instituto Militar de Engenharia, Praça Gen. Tibúrcio, 80 - Urca, Rio de Janeiro, RJ 22290-270 Brazil; 2 Instituto de Química, Universidade Federal Rural do Rio de Janeiro (UFRRJ), Seropédica, Rio de Janeiro, RJ 23890-000 Brazil

**Keywords:** Dye-sensitized solar cells (DSSCs), D-π-A-π-A quinoxaline-based dyes, Charge transfer, Photovoltaic performance, Electronic excited states

## Abstract

**Context:**

The design of efficient metal-free organic dyes is crucial for improving the performance of dye-sensitized solar cells (DSSCs). In this work, we investigated the influence of different donor groups on the electronic, photophysical, and photovoltaic properties of two D-*π*-A-*π*-A quinoxaline-based dyes, QX-DMA and QX-TPA. Density functional theory (DFT) and time-dependent DFT (TDDFT) calculations with solvation in tetrahydrofuran (THF) were employed to examine structure–property relationships. The optimized geometries exhibit high planarity along the conjugated backbone, favoring *π*-conjugation and intramolecular charge transfer. The results indicate that QX-TPA exhibits superior photovoltaic parameters, including a higher electron injection efficiency, greater regeneration driving force, and lower charge recombination, which are attributed to the extended electronic delocalization promoted by the triphenylamine (TPA) group. Electronic analysis shows that the lowest singlet excited state (${S}_{1}$) is a bright HOMO → LUMO transition with pronounced electron–hole separation. Owing to its electron-donating group and more extended *π*-delocalization, QX-TPA displays enhanced charge-transfer character, larger reorganization energy, and a significantly longer excited-state lifetime, indicating improved charge stabilization and slower recombination rate compared with QX-DMA. These molecular properties suggest enhanced photovoltaic performance, with QX-TPA-based DSSCs achieving nearly twice the power conversion efficiency of QX-DMA devices, mainly due to a higher short-circuit current density.

**Methods:**

The geometrical and electronic properties of all systems were investigated using density functional theory with the B3LYP exchange–correlation functional combined with the 6-311G(d,p) basis set. The functionals B3LYP-D3(BJ), CAM-B3LYP, *ω*B97X, and M06-2X were also tested. Time-dependent DFT calculations were performed at the same level of theory to describe the first ten singlet excited states (${S}_{1} - {S}_{10}$). THF solvation effects were accounted for using the conductor-like polarizable continuum model (CPCM). All calculations were carried out using the ORCA 6.0 program package. Charge-transfer properties were analyzed using the TheoDORE program, whereas molecular orbital fragmentation analysis employed the Multiwfn (version 3.8) program package.

**Supplementary Information:**

The online version contains supplementary material available at https://doi.org/10.1007/s00894-026-06944-9.

## Introduction

The growing global energy demand and the environmental impacts resulting from fossil fuel combustion make the transition to renewable sources essential. The burning of these resources releases ${CO}_{2}$ and intensifies the greenhouse effect, whereas solar energy, clean and abundant, does not generate emissions during its conversion into electricity, making it one of the most promising routes toward sustainable development [1–5].

Among emerging photovoltaic technologies, dye-sensitized solar cells (DSSCs) stand out as a highly viable alternative to traditional silicon-based devices, combining low production costs, straightforward fabrication processes, and competitive power conversion efficiencies (PCE). Since their pioneering introduction [6], the quest for higher efficiencies has focused on the optimization of co-sensitized dyes and solid-state electrolytes. Although isolated milestones approaching 15% or utilizing tandem configurations have been achieved under laboratory conditions under standard AM 1.5G solar illumination (100 mW cm⁻^2^) [6–10], the certified standard efficiency for single cells at a commercial scale remains around 13%. This continuous progress has been driven primarily by the rational molecular engineering of novel organic sensitizers and the development of advanced redox mediator systems. Furthermore, under indoor lighting conditions typically measured using artificial light-emitting diodes (LEDs) or fluorescent lamps at low illuminance, DSSCs have demonstrated even more impressive efficiencies ranging from 28 to over 36% [11–13]. These outstanding results under low-intensity environments are intrinsically linked to the excellent spectral match between the absorption profiles of organic dyes and the emission spectra of artificial light sources. Coupled with continuous advances in structural material design and rigorous interfacial engineering among the device components, these improvements position DSSCs as highly promising candidates for both outdoor energy generation and diffuse ambient light-harvesting applications [12].

A typical DSSC consists of five components: a transparent conductive substrate, usually glass coated with fluorine-doped tin oxide (FTO), responsible for electrical conduction and mechanical support [12]; a mesoporous film of a semiconductor metal oxide, such as nanocrystalline ${TiO}_{2}$, which acts as an electron collector and transporter [14]; a dye sensitizer, responsible for light absorption and electron injection into the semiconductor; an electrolyte, which transports holes and completes the electrical circuit [15]; and a counter electrode, typically platinum or carbon on FTO, which catalyzes the regeneration of the redox mediator [16].

The overall performance of the cell strongly depends on the dye sensitizer, which is responsible for both light harvesting and charge transfer. An ideal photosensitizer should meet several criteria, namely: (a) a broad absorption spectrum and a high molar extinction coefficient [16]; (b) steric features that reduce aggregation and charge recombination on the ${TiO}_{2}$ surface [17]; (c) properly aligned energy levels, with the LUMO positioned above the ${TiO}_{2}$ conduction band at − 4.0 eV, ensuring efficient electron injection [18], and the HOMO located below the redox potential of the electrolyte (− 4.8 eV), allowing dye regeneration [8, 19]; and (d) sufficient photochemical and thermal stability to guarantee device durability [8, 20–22].

In recent years, metal-free organic dyes have gained prominence as alternatives to ruthenium complexes due to their high molar absorptivity, low cost, versatile chemical structures, and environmental compatibility [23]. These sensitizers generally exhibit a D-*π*-A architecture, comprising an electron-donating group (D), a conjugated *π*-bridge, and an electron-withdrawing/anchoring group (A) [24]. Structural modifications of this basic design have led to the development of extended D-*π*-A-*π*-A systems, which enhance charge separation, broaden the absorption spectrum, and promote better electronic coupling between donor and acceptor units [25, 26]. Furthermore, the introduction of a second *π*-conjugated bridge can reduce electron recombination and increase the lifetime of the excited states, thereby enhancing electron injection into the semiconductor [27]. However, the length and chemical nature of the *π*-bridge must be carefully optimized to avoid weakening the electronic coupling between the donor and acceptor moieties [28–31].

Among the various structural blocks employed in organic dyes, quinoxaline stands out as an effective electron-accepting unit due to its electron-deficient character, structural rigidity, and ability to modulate HOMO/LUMO energy levels. The introduction of appropriate donor groups is crucial for fine-tuning the electronic and photophysical properties of these dyes, thereby directly influencing charge-transfer processes and, consequently, photovoltaic performance [32–34].

Our research group has investigated various conjugated systems of interest to organic photovoltaics using Density Functional Theory (DFT), Time-Dependent Density Functional Theory (TDDFT), and the ab initio wave function ADC(2) (Algebraic Diagrammatic Construction to second order) [35–42]. In this work, the focus is on quinoxaline derivatives.

Although triphenylamine (TPA) is widely recognized as a strong electron-donating unit in organic dyes, the experimental data reported for this molecular framework reveal that the QX-DMA dye exhibits a 10 nm bathochromic shift of the intramolecular charge transfer (ICT) band compared to QX-TPA, indicating a more stabilized excited state and therefore a stronger effective donor ability in this system [43]. On the other hand, QX-TPA displays a more intense absorption, which has been attributed to the enhanced conjugation provided by the phenyl substituents and to the nonplanar helical conformation of the TPA core, which suppresses dye aggregation and increases the molar extinction coefficient [44–46].

**Fig. 1:**
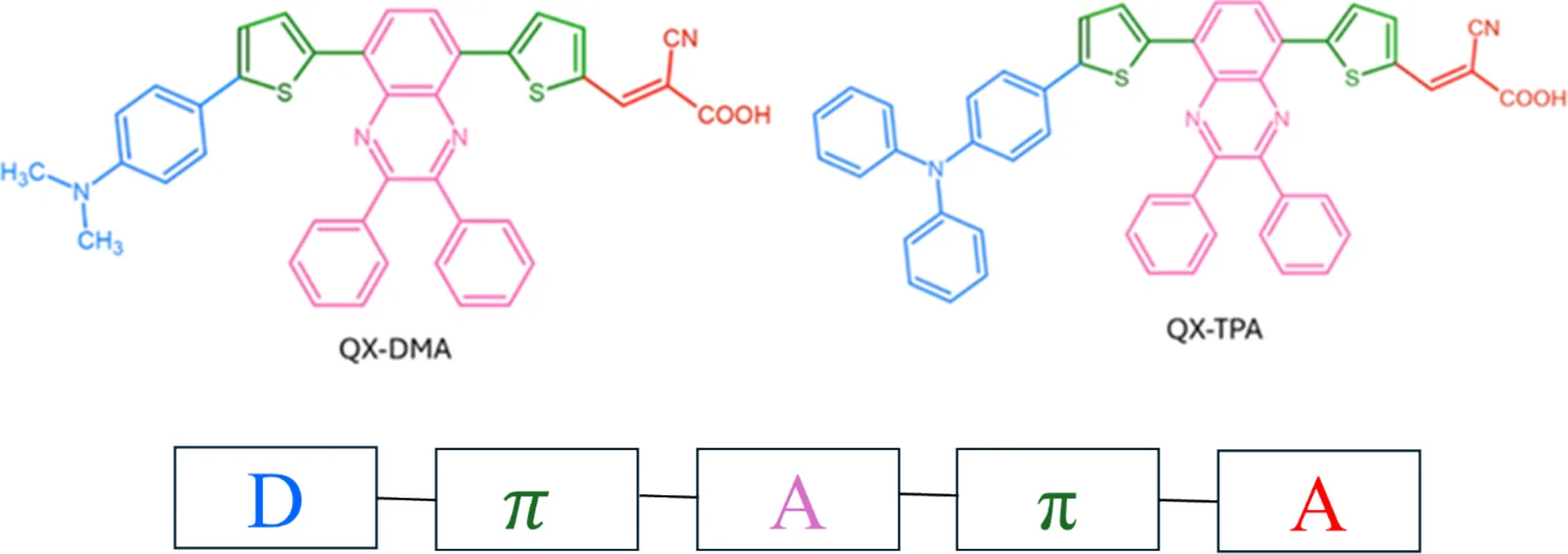
Chemical structures of the QX-DMA and QX-TPA dyes and their D-*π*-A-*π*-A molecular configuration. The donor (D), acceptor units (A1 and A2), and π-bridge moieties are indicated in different colors

In this work, we investigate the influence of different arylamine donor groups, namely *N*,*N*-dimethylaniline (DMA) and triphenylamine (TPA), on the electronic, photophysical, and photovoltaic properties of quinoxaline-based D-*π*-A-*π*-A organic dyes using density functional theory (DFT) and time-dependent density functional theory (TDDFT). The studied dyes, denoted QX-DMA and QX-TPA, are illustrated in Fig. 1. The dyes share a 2,3-diphenylquinoxaline-fused conjugated backbone (first electron acceptor, (A1); shown in pink). This backbone incorporates thiophene units acting as *π*-bridges (shown in green), linking the donor moieties (shown in blue) to the cyanoacrylic group (second electron acceptor and anchoring unit, (A2); shown in red), which anchors the dye to the semiconductor surface.

## Theoretical methods

### Computational details

All calculations were performed using the ORCA 6.0.1 software package [47]. Equilibrium geometries of QX-DMA and QX-TPA were fully optimized using B3LYP/6-311G(d,p) [48–50] without imposing symmetry constraints. Harmonic vibrational frequency analyses confirmed the lack of imaginary frequencies; thus the optimized structures correspond to true minima on the potential energy surface. Frontier Molecular Orbital (FMO) energies (${E}_{HOMO}$ and ${E}_{LUMO}$) and other electronic properties relevant to photovoltaic devices were obtained using the optimized geometries. Geometry optimizations were initially performed using the B3LYP functional. Subsequently, Grimme’s D3BJ dispersion correction was included to ensure highly accurate structural parameters by accounting for intramolecular non-covalent interactions [51]. The Pople 6-311G(d,p) basis set family [46, 48, 52] was adopted because it yields a robust description of the electronic density for neutral, covalently bonded molecules containing heteroatoms [48]. The inclusion of polarization functions (d,p) is essential for accurately describing charge redistribution and orbital distortion during chemical bonding processes [53]. For the electronic characterization of the ground state, the frontier molecular orbitals (HOMO and LUMO) were analyzed using the Multiwfn software [54].

The converged geometries of the cationic and anionic species were fully relaxed to determine the adiabatic reorganization energies. Reactivity descriptors, including the chemical potential**,** chemical hardness**,** electronegativity, and electrophilicity index**,** were computed from the adiabatic ionization potentials and electron affinities.

To further investigate the photophysical properties of the dyes, single-point calculations were carried out on the geometries previously optimized at the B3LYP/6-311G(d,p) level. The B3LYP structures were retained for these calculations because the inclusion of D3(BJ) dispersion corrections resulted in marginal geometric variations, as discussed in detail in the “Ground-state geometries of the dyes” Section. The ten lowest singlet excited states were determined through Time-Dependent Density Functional Theory (TDDFT) using the Tamm–Dancoff Approximation (TDA). We also evaluated the performance of five density functionals: B3LYP, CAM-B3LYP [55], *ω*B97X [56], PBE [49], and M06-2X [57]. Solvent effects were incorporated using the implicit solvation model Integral Equation Formalism Polarizable Continuum Model (IEF-CPCM) [58, 59] for tetrahydrofuran (THF), characterized by a dielectric constant (ε) of 7.42.

The radiative lifetimes were estimated within the vertical Franck–Condon approximation [60–62] using excitation energies and oscillator strengths calculated at the ground-state equilibrium geometry [33, 63, 64]. In this framework, the computed values should be regarded as comparative indicators of radiative decay propensity rather than quantitative fluorescence lifetimes, since excited-state structural relaxation and vibronic contributions are not explicitly considered. For the investigated quinoxaline-based dyes, the optimized B3LYP and B3LYP-D3(BJ) geometries exhibit only minor structural differences, indicating a rigid and highly conjugated molecular framework. Therefore, the electronic transition properties are expected to be only weakly affected by small geometric variations, supporting the use of the ground-state geometry as a computationally efficient approximation for estimating radiative lifetimes.

For a more accurate representation of the charge-transfer character of the electronic excitations and the spatial distribution of electron–hole pairs, Natural Transition Orbitals (NTOs) and intramolecular charge-transfer descriptors were obtained using the TheoDORE package [65]. To ensure computational setup compatibility and reliability, the one-particle transition density matrices 1-TDMs and excitation amplitudes were directly parsed from the ORCA TD-DFT output files into the TheoDORE interface. This workflow allows for a rigorous quantitative analysis of exciton wave functions and the calculation of charge-transfer numbers via a fragment-based decomposition of the transition density, following the well-established methodology described by Plasser and Lischka [61, 62, 65, 66].

**Fig. 2:**
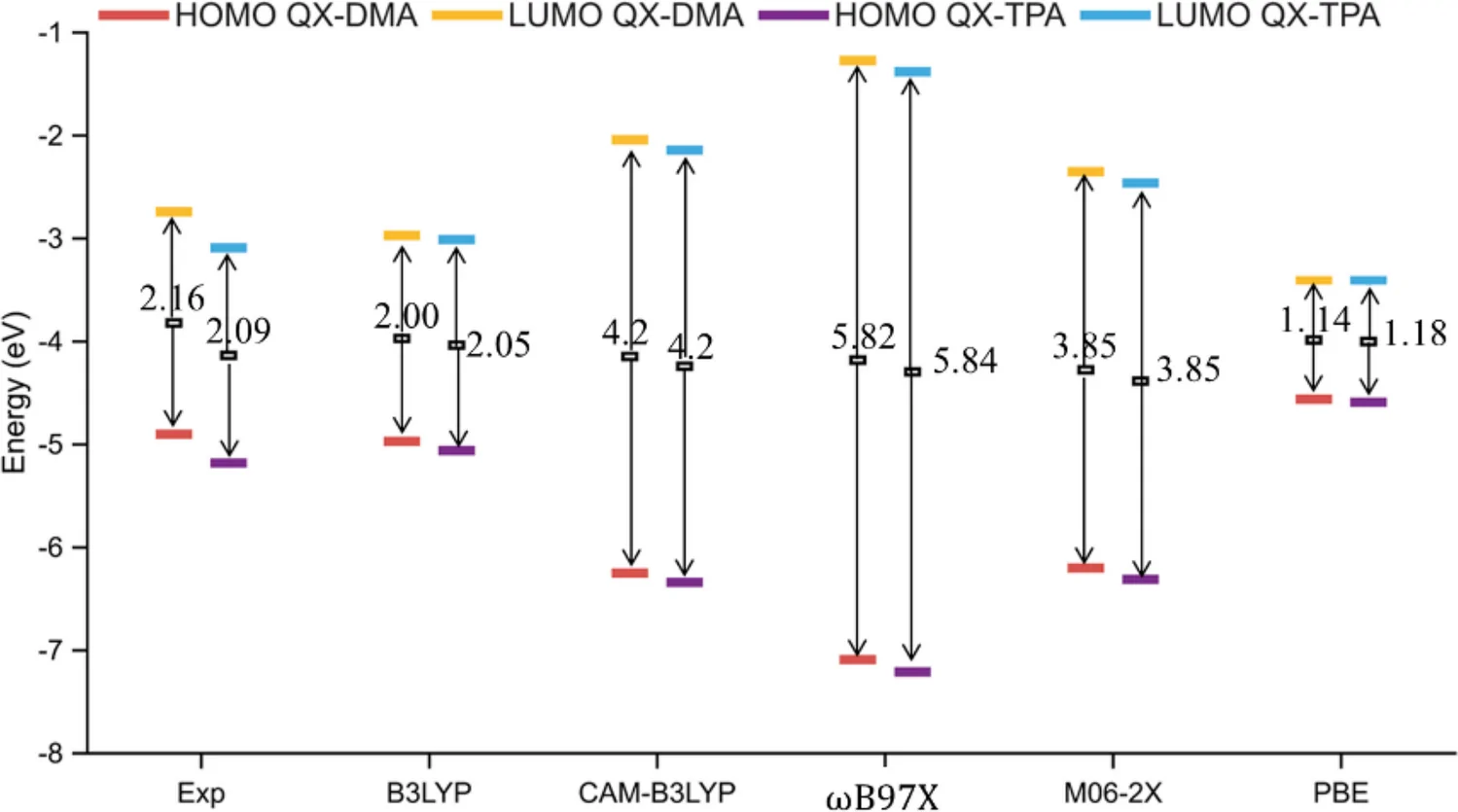
HOMO/LUMO energy levels and band gaps of QX-DMA and QX-TPA dyes calculated using different exchange–correlation functionals (B3LYP, CAM-B3LYP, ω B97X, M06-2X, and PBE) compared with experimental data

This approach enables a direct comparison of ground and excited-state electronic properties predicted by different functionals, including HOMO/LUMO energies, band gaps, and vertical excitation energies without re-optimizing the molecular structures**,** thereby isolating the intrinsic effect of the exchange–correlation functional on the results. As illustrated in Fig. 2, this approach provides a consistent basis for evaluating how each functional describes the dyes’ electronic structure. The computed HOMO and LUMO energies and the corresponding energy gaps are summarized in Table S1 in the Supporting Information. Among the investigated functionals, B3LYP exhibited the closest agreement with the experimental absorption data, whereas the long-range corrected and meta-GGA functionals showed larger deviations. All pictorial representations of the data were produced with the MATLAB software [67].

To systematically evaluate the performance of the five functionals, TDDFT calculations were initially carried out in the gas phase and subsequently refined with the inclusion of solvent effects. In the gas phase (Table S2, Supporting Information), the B3LYP and PBE functionals significantly overestimate the ${\lambda}_{max}$ values, whereas CAM-B3LYP and ω B97X tend to underestimate them. Upon inclusion of solvent effects in tetrahydrofuran (THF) solution (Table S3, Supporting Information), the deviations relative to experimental data [68] are reduced for most functionals. This improvement is particularly noticeable for CAM-B3LYP and M06-2X, which exhibit the smallest wavelength shifts ($\Delta {\lambda}_{max}$) for QX-DMA, the shifts are 40.5 nm and 32 nm, respectively, while for QX-TPA, the corresponding values are 43.8 nm and 38 nm.

Regarding the frontier molecular orbital (FMO) energies, the B3LYP functional showed excellent agreement with experimental data, as evidenced by the small theoretical–experimental energy differences summarized in Table S4. For QX-DMA, the differences are ${\Delta E}_{HOMO} = -0.07 \mathrm{e}\mathrm{V}$ and ${\Delta E}_{LUMO} = -0.23 \mathrm{e}\mathrm{V}$, whereas for QX-TPA the corresponding values are ${\Delta E}_{HOMO} = 0.12$ eV and ${\Delta E}_{LUMO} = -0.08 \mathrm{e}\mathrm{V}$. Therefore, although B3LYP exhibits noticeable deviations regarding the maximum absorption wavelengths, it yields the most accurate frontier molecular orbital energies among the evaluated functionals. Since the key photovoltaic and thermodynamic descriptors investigated in the subsequent sections are primarily derived from ${E}_{HOMO}$ and ${E}_{LUMO}$ values, the selection of the B3LYP functional is appropriate in this case for the remaining electronic structure analyses.

### Optoelectronic properties

The photovoltaic performance of a dye-sensitized solar cell can be quantified through the power conversion efficiency (PCE), which depends on fundamental device parameters, including the short-circuit current density ${( J}_{SC})$, the open-circuit voltage ${V}_{oC}$ and the fill factor (FF) [18, 28, 69]:

1 $$PCE=\frac{\left(V_{oC}\times J_{sC}\times FF\right)}{P_{inc}}$$

Power conversion efficiency is one of the most important parameters governing the overall performance of dye-sensitized solar cell (DSSC) devices. However, the direct theoretical evaluation of PCE at the molecular level remains a significant challenge, since device efficiency inherently depends on multiple macroscopic and experimental factors that cannot be fully captured by isolated molecular calculations [70–72]. In this context, theoretical studies commonly rely on the analysis of molecular descriptors such as frontier molecular orbital energies, light-harvesting efficiency (*LHE*), driving forces for electron injection and dye regeneration, and parameters associated with intramolecular charge transfer (ICT) [67]. These descriptors are closely correlated with key experimental photovoltaic quantities, such as short-circuit current density (${J}_{SC}$) and open-circuit voltage (${V}_{OC}$), thereby enabling the reliable prediction of relative trends in the photovoltaic performance of the investigated dyes [70, 72, 73].

The open-circuit voltage $${V}_{OC}$$ can be evaluated using Eq. (2) [74, 75], which is the energy difference between the dye’s LUMO energy ($${E}_{LUMO}$$) and the conduction band of titanium dioxide ($${E}_{CB}^{{TiO}_{2}} = -4.0 eV)$$ [76], representing the theoretical maximum energy available for electron transfer within the device:

2 $$V_{OC}=E_{LUMO}-E_{CB}^{{TiO}_2}$$

The short-circuit current density can be determined using Eq. (3) [77]:

3 $$J_{SC}=e\int LHE\left(\lambda\right)\Phi_{inj}\eta_{reg}\eta_{coll}\varphi_{ph.AM1.5G}d\lambda$$

where LHE is the light-harvesting efficiency at a given wavelength λ, ${\Phi}_{inj}$ is the electron injection efficiency calculated from the driving force for electron injection (${\Delta \mathrm{G}}_{inject}$) defined below; ${\eta}_{coll}$ (charge collection efficiency) and ${\eta}_{reg}$(dye regeneration efficiency) are assumed to be unity; this yields the theoretically maximum short-circuit current density, reflecting the overall light-harvesting capabilities of the dyes [78–81]. ${\varphi}_{ph.AM1.5G}$ corresponds to the photon flux under AM1.5G solar irradiation [82], evaluated over the absorption spectrum constructed from the computed excitation wavelengths (λ) and oscillator strengths (f). The Python script used to calculate the ${J}_{SC}$ values is provided in the Supporting Information.

The light-harvesting efficiency LHE can be determined according to the following expression [83]:

4 $$LHE=1-10^{-f}$$

where f is the oscillator strength associated with the strongest electronic transition. A higher f value reflects stronger light absorption and, consequently, a greater ability of the dye to harvest photons, which contributes directly to an enhanced ${J}_{SC}$ value [84]. The fill factor FF can be computed using the empirical expression [84, 85]:

5 $$FF=\frac{\frac{e\,V_{OC}}{k_BT}-\mathit{ln}\left(\frac{e\,V_{OC}}{k_BT}+0.72\right)}{\frac{e\,V_{OC}}{k_BT}+1}$$

where e is the elementary charge, ${k}_{B}$ is the Boltzmann constant, and T is the absolute temperature [84, 85].

The driving force for electron injection (${\Delta \mathrm{G}}_{inject}$) quantifies the thermodynamic feasibility of transferring an electron from the excited dye to the conduction band of ${TiO}_{2}$ [76, 86, 87]. It can be expressed as:

6 $${\Delta\mathrm{G}}_{inject}=E_{dye}^\ast-E_{CB}^{{TiO}_2}$$

where ${E}_{CB}^{{TiO}_{2}}$ is the conduction band value of ${\mathrm{T}\mathrm{i}\mathrm{O}}_{2}$($-4.0 \mathrm{e}\mathrm{V}$ [63]), and ${E}_{dye}^{*}$ is the excited-state oxidation potential of the dye. The latter can be computed by the following relationship [88]:

7 $$E_{dye}^\ast=E_{dye}-E_{0-0}$$

where ${E}_{dye}$ is the oxidation potential of the dye in the ground state, determined from the negative of the highest occupied molecular orbital energy (${E}_{HOMO}$), and ${E}_{0-0}$ is the lowest vertical electronic excitation energy, corresponding to ${\lambda}_{max}$ [89].

The dye regeneration free energy ${\Delta G}_{regen}$, a key factor determining DSSC efficiency, is calculated as [90–92]:

8 $${\Delta G}_{regen}=E_{I^-/I_3^-}-E_{dye}$$

where ${E}_{{I}^{-}/{I}_{3}^{-}}$ is the redox potential of the electrolyte. Negative values of ${\Delta G}_{regen}$ indicate that regeneration is thermodynamically favorable, ensuring continuous photovoltaic conversion. The ${E}_{{I}^{-}/{I}_{3}^{-}}$ reference value is –4.8 eV [93, 94]. The Gibbs free energy change for the charge recombination process ${\Delta G}_{recomb}$, where an electron injected into the TiO_2_ conduction band moves backward to recombine with the oxidized dye’s highest occupied molecular orbital is given by:

9 $${\Delta G}_{recomb}=E_{CB}^{{TiO}_2}-E_{HOMO}$$

The variation in dipole moment upon electronic excitation was computed from the vector difference between the excited-state and ground-state dipole moments according to [95, 96]:

10 $$\Delta\upmu = \sqrt{{\mu}_{x}^{{S}_{1}}-{\mu}_{x}^{{S}_{0}}+{\mu}_{y}^{{S}_{1}}-{\mu}_{y}^{{S}_{0}}+{\mu}_{z}^{{S}_{1}}-{\mu}_{z}^{{S}_{0}}} (10)$$

where ${\mu}_{x}$, ${\mu}_{y}$, and ${\mu}_{z}$ correspond the Cartesian components of the dipole moment vector for the ground (${S}_{0}$) and excited (${S}_{1}$) electronic states. The calculated $\Delta \mu$ values were used to evaluate the degree of electronic redistribution and intramolecular charge-transfer character upon photoexcitation [95, 96].

Reorganization energies account for the structural and electronic relaxation of the dye during charge transfer, which are crucial for estimating electron-transfer rates according to Marcus theory [97]. The total reorganization of energy is expressed as the sum of two terms [97, 98].

11 $$\lambda_{total}=\lambda_e+\lambda_h$$

where ${\lambda}_{h}$ is the hole reorganization energy:

12 $${\lambda}_{h}=\left({E}_{0}^{+} - {E}_{0}\right) +\left({E}_{+}^{0}-{E}_{+}\right)$$

where ${E}_{0}^{+}$ is the energy of the neutral dye calculated at the cationic geometry and ${E}_{0}$ corresponds to the energy of the neutral dye at its optimized geometry. Similarly, ${E}_{+}^{0}$ refers to the energy of the cation evaluated at the neutral geometry, while ${E}_{+}$ represents the energy of the cation in its optimized geometry. Similarly, the electron reorganization energy ${\uplambda}_{{\boldsymbol{e}}}$ is given by [97, 98]:

13 $${\uplambda}_{{\boldsymbol{e}}}=\left({E}_{0}^{-} - {E}_{0}\right) +\left({E}_{-}^{0}-{E}_{-}\right)$$

where ${E}_{0}^{-}$ is the energy of the neutral dye calculated at the anion geometry and ${E}_{0}$ represents the energy of the neutral dye in its equilibrium structure. ${E}_{-}^{0}$ is the energy of the anion calculated at the neutral geometry, whereas ${E}_{-}$ is the energy of the anion at its fully optimized geometry.

The radiative excited-state lifetime τ is a fundamental photophysical parameter that quantifies the probability of spontaneous decay from the singlet excited state to the ground state [33, 94]. Dyes with longer excited-state lifetimes tend to suppress charge recombination, thereby enhancing electron injection efficiency [76] and improving the overall power conversion efficiency (PCE) of dye-sensitized solar cells. In this work, the lifetime values were determined from TDDFT calculations employing the vertical excitation energy (E) and oscillator strength (f) of the lowest singlet excited state **(**${S}_{0}$** →** ${S}_{1}$ transition) [99], according to [76, 100]:

14 $$\tau =\frac{1.499}{f {E}^{2}}$$

where 1.499 is a constant that incorporates the relevant physical constants and unit conversions, with E is expressed in eV, f is the dimensionless oscillator strength, and τ is obtained directly in nanoseconds [101].

Charge-transfer properties were determined using TheoDORE, a computational package for excited-state analysis that evaluates electronic transitions in terms of electron–hole interactions, based on the one-electron transition density matrix (1TDM) between the ground and excited states [65, 66, 102]:

15 $$\gamma \left({r}_{h} ,{r}_{e}\right)= \int ...\int {\Psi}_{0} \,\left({r}_{h}, {r}_{2},...{r}_{n}\right){ {\Psi}_{1}\left({r}_{e}{ ,r}_{2}...{r}_{n}\right) dr}_{2} ...{dr}_{2}$$

where ${\Psi}_{0}$ and ${\Psi}_{1}$ are the ground and excited state wavefunctions, respectively. The coordinates ${r}_{h}$ and ${r}_{e}$ correspond to the hole and electron, originating from ${\Psi}_{0}$ and ${\Psi}_{1}$, respectively. Using the 1TDM, each excitation can be decomposed into local excitation (LE) and charge-transfer ($\mathrm{C}\mathrm{T}$) contributions. These contributions are quantified by the charge-transfer numbers (${\Omega}_{AB}$) computed by [65, 66, 102]:

16 $$\Omega_{AB}=\int\limits_A\int\limits_B\left|\gamma\left(r_h,r_e\right)\right|^2{dr}_ed{r}_h$$

where the hole is restricted to fragment *A* and the electron to fragment *B*. These numbers quantify the extent of electron–hole correlation between different fragments of the system.

The exciton size $\widetilde{{d}_{ex}}$ was calculated to quantify the average spatial separation between the electron and hole [65, 66, 102]:

17 $$\widetilde{{d}_{ex}} =\sqrt{{\Omega }^{-1}{\Omega}_{MN}{d}_{MN }^{2}}$$

where ${\Omega}_{MN}$ is the charge-transfer number computed between atoms (or fragments) M and N, ${d}_{MN}$ is the distance between these atoms and Ω is the normalization factor defined as the sum over all ${\Omega}_{MN}$ elements. This descriptor corresponds to the root-mean-square electron–hole separation (RMSeh). Values below approximately 4 Å indicate local excitations, whereas larger values represent charge-transfer effects [65, 102].

Natural Transition Orbital (NTO) analysis was carried out to assess the nature of the excited states. The NTO participation ratio (${PR}_{NTO}$) was evaluated by the following equation [65, 66, 102]:

18 $${PR}_{NTO}= \frac{{\left(\sum_{t}{\lambda}_{t}\right)}^{2}}{\sum_{t}{\lambda}_{t}^{2}}$$

where ${\lambda}_{t}$ are the eigenvalues obtained from the singular value decomposition of the 1TDM. This descriptor quantifies the effective number of NTO pairs contributing to a given excitation. A value close to 1 indicates that the transition is dominated by a single NTO pair [65, 66, 102].

The combined analysis of these descriptors provides a comprehensive picture of the excited-state states, enabling the distinction between localized excitations and long-range charge-transfer processes in the studied dyes.

### Molecular reactivity descriptors

Vertical ionization potentials (IP) and vertical electron affinities (EA) are crucial parameters for characterizing the electronic structure of molecular systems [103]. The IP is the minimum energy required to remove an electron from a neutral molecule, while the EA is the energy released upon adding an electron. In the adiabatic approach, the IP is calculated as the total energy difference between the optimized cation (anion for *EA*) and the optimized neutral molecule according to Eqs. (19) and (20) [103]:

19 $$IP ={(E}_{cation}) -({E}_{neutral})$$

20 $$EA ={(E}_{neutral}) -({E}_{anion})$$

The electronegativity **(**χ), representing the tendency of a molecule to attract electrons, is defined as the average of the ionization potential (*IP*) and electron affinity (*EA*) [104, 105]:

21 $$\chi =\frac{IP +EA}{2}$$

The chemical hardness (η)**,** which indicates the resistance of a molecule to changes in its electronic distribution, is expressed as [106, 107]:

22 $$\upeta =\frac{IP - EA}{2}$$

From these parameters, the electrophilicity index (ω), quantifying the ability of a molecule to accept electrons from a donor, can be calculated [104, 107]:

23 $$\omega = \frac{(\mathrm{I}\mathrm{P}+\mathrm{E}\mathrm{A}{)}^{2}}{8\upeta }$$

24 $${\omega }^{+}= \frac{(\mathrm{I}\mathrm{P}+3\mathrm{A}{)}^{2}}{16\upeta (\mathrm{I}\mathrm{P}-\mathrm{E}\mathrm{A})} ,\, {\omega }^{-}= \frac{(\mathrm{I}\mathrm{P}+\mathrm{E}\mathrm{A}{)}^{2}}{16\upeta (\mathrm{I}\mathrm{P}-\mathrm{E}\mathrm{A})}$$

The parameters ${\omega }^{-}$ and ${\omega }^{+}$ quantify the ability of the dyes to donate or accept electronic charge, respectively. For a good photovoltaic performance, higher values of these indices are generally desirable, as they indicate a greater propensity to participate in electron-transfer processes [108].

## Results and discussion

### Ground-state geometries of the dyes

**Scheme 1:**
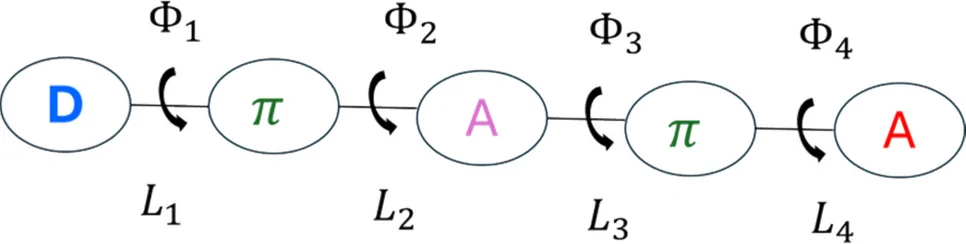
Schematic structure of the investigated D-*π*-A-*π*-A dyes. ${\Phi}_{1}$, ${\Phi}_{2}$, ${\Phi}_{3}$, and ${\Phi}_{4}$, are the dihedral angles, and ${L}_{1}$, ${L}_{2}$, ${L}_{3}$ and ${L}_{4}$, the bond lengths

The B3LYP/6-311G(d,p) optimized geometries of the QX-DMA and QX-TPA dyes are shown in Fig. S1 (Supporting Information), while the corresponding bond lengths $({L}_{1} - {L}_{4}$) and dihedral angles (${\Phi}_{1}- {\Phi}_{4}$) calculated for both the B3LYP/6-311G(d,p) and B3LYP-D3(BJ)/6-311G(d,p) geometries are summarized in Table 1. Scheme 1 depicts these angles and bond lengths.

**Table 1:** Results obtained using the B3LYP and the dispersion-corrected B3LYP-D3(BJ) geometries: selected bond lengths L (in Å) and dihedral angles Φ (in °) for the QX-DMA and QX-TPA dyes

Dyes	${L}_{1}$	${L}_{2}$	${L}_{3}$	${L}_{4}$	${\Phi}_{1}$	${\Phi}_{2}$	${\Phi}_{3}$	${\Phi}_{4}$
QX-DMA–B3LYP	1.46	1.45	1.45	1.42	− 179.8	178.5	179.5	− 179.8
QX-TPA–B3LYP	1.46	1.45	1.45	1.42	− 179.9	177.0	176.9	179.7
QX-DMA–B3LYP-D3	1.45	1.45	1.45	1.42	− 179.9	179.4	179.7	− 179.9
QX-TPA–B3LYP-D3	1.45	1.45	1.45	1.42	179.9	179.2	179.7	179.9

The electronic and optical properties of the D-*π*-A-*π*-A QX-DMA and QX-TPA dyes are strongly influenced by the planarity of their structures [109]. As shown in Table 1, the calculated inter-ring bond lengths along the conjugated backbone vary between 1.42 and 1.46 Å for both dyes. These bond lengths, being significantly shorter than a typical single C–C bond (~ 1.53 Å) [110], confirm a strong resonance character and effective *π*-electron delocalization between the *π*-spacers and the acceptor groups. Furthermore, the calculated dihedral angles across the D-*π*-A-*π*-A sequence remain remarkably close to 180° (ranging from 176.9 to 179.9°), demonstrating that the donor, *π*-linkers, and acceptor units are nearly coplanar. This structural arrangement ensures an extended π-conjugation along the entire molecular bridge, and these minor deviations from planarity do not significantly hinder the intramolecular charge transfer (ICT) from the donor to the terminal acceptor [111]. Interestingly, although triphenylamine (TPA) derivatives are generally known for their non-coplanar, propeller-like nature [110, 112], the planarity of QX-TPA is largely preserved by the rigid D-*π*-A-*π*-A architecture. Furthermore, the inclusion of Grimme’s dispersion corrections in the B3LYP-D3(BJ) calculations yields only marginal geometric variations with bond length differences of at most 0.01 Å and negligible shifts in dihedral angles, thereby confirming the high structural rigidity of both systems (Table S5). Overall, this highly planar arrangement minimizes structural reorganization and may facilitate efficient excited-state electron injection into the ${TiO}_{2}$ conduction band, enhancing visible-light absorption, and optimizing dye performance in DSSCs.

### Frontier molecular orbitals (FMOs)

**Fig. 3:**
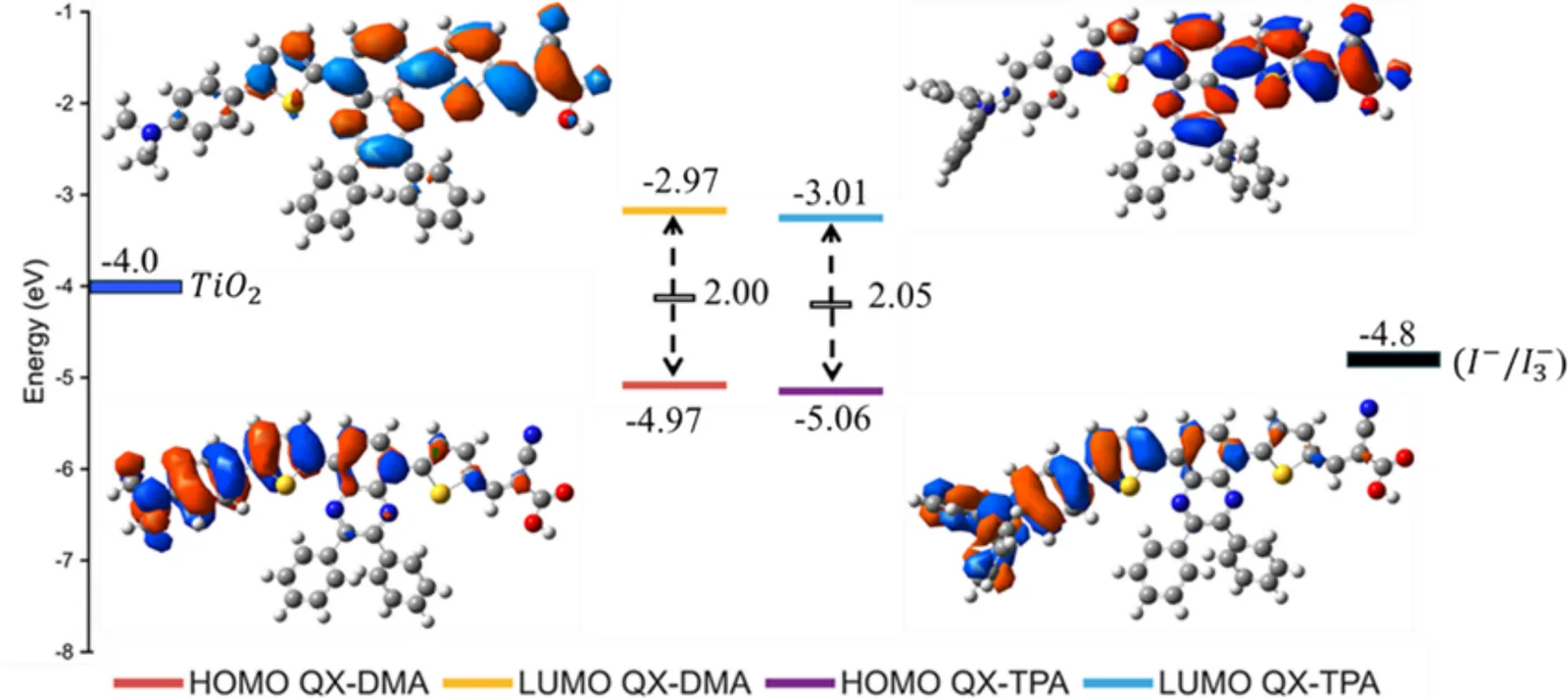
B3LYP/6-311G(d,p) energy level diagram of the QX-DMA and QX-TPA dyes, showing the HOMO and LUMO orbital levels compared with the conduction band of ${TiO}_{2}$ and the redox potential of the ${(I}^{-}/{I}_{3}^{-})$ couple values

The spatial distribution of the frontier molecular orbital (FMOs) is related to the charge-transfer characteristics of the dyes. By comparing the relative positions of the HOMO and LUMO energy levels, the system’s ability to gain or lose electrons during redox processes can be qualitatively assessed [113, 114]. For charge injection from the photoexcited dye to occur, the ${E}_{LUMO}$ of the dye must be energetically positioned above the conduction band (CB) of the ${TiO}_{2}$ semiconductor (− 4.0 eV), providing the driving force required for electron transfer [115]. Furthermore, for efficient dye regeneration via electron donation from the electrolyte, the ${E}_{HOMO}$ of the dye must lie below the redox potential of the iodide/triiodide (${I}^{-}/{I}_{3}^{-}$) electrolyte (− 4.8 eV) [116]. Figure 3 illustrates the molecular energy levels of the QX-DMA and QX-TPA dyes, as well the corresponding molecular orbitals surfaces, highlighting the influence of the donor groups (DMA and TPA) on the HOMO and LUMO energies and on the HOMO–LUMO energy gap. Overall, both dyes exhibit suitable HOMO and LUMO energy levels, indicating their potential as effective photosensitizers for dye-sensitized solar cells.

As shown in Fig. 3, the QX-DMA dye exhibits a higher HOMO energy level (− 4.97 eV) than the QX-TPA dye (− 5.06 eV). This behavior can be attributed to the greater electron-donating character of the dimethylamine (DMA) group relative to that of triphenylamine (TPA). Strong electron-donating groups tend to raise the HOMO energy, promoting greater electronic delocalization along the *π*-conjugated system and, consequently, contributing to a reduction of the energy gap [117]. In this work, the higher HOMO energy of QX-DMA results in an energy gap of 2.00 eV, favoring lower-energy electronic transitions and suggesting that its light absorption may extend to longer wavelengths compared to QX-TPA.

However, it is crucial to distinguish this thermodynamic inductive effect from the spatial intramolecular charge-transfer efficiency upon photoexcitation. For both dyes, the HOMO is predominantly localized over donor moieties *N*,*N*-dimethylaniline (DMA) in QX-DMA and triphenylamine (TPA) in QX-TPA with partial delocalization along the adjacent *π*-conjugated bridge. According to the Ros-Schuit (SCPA) [54, 118] orbital decomposition analysis (Table 2), the donor fragment (D) contributes 61.46% to the HOMO for QX-DMA and 69.50% for QX-TPA. In contrast, the LUMO is mainly distributed over the quinoxaline-based acceptor segment and the cyanoacrylic acid anchoring group through the conjugated *π*-bridge, exhibiting the characteristic spatial separation between donor and acceptor regions that promotes intramolecular charge transfer upon photoexcitation [89].

**Table 2:** Orbital decomposition analysis (%) by fragment for QX-DMA and QX-TPA dyes

Dyes	Orbital	*D* (%)	${\pi}_{1}$(%)	A1 (%)	${\pi}_{2}$(%)	A2 (%)
QX-DMA	HOMO	61.46	24.00	10.27	3.70	2.10
QX-DMA	LUMO	28.11	7.53	35.00	22.61	25.80
QX-TPA	HOMO	69.50	17.90	8.12	2.85	1.64
QX-TPA	LUMO	2.40	7.82	42.73	22.11	24.95

The electron density partitioning data obtained from the SCPA analysis confirms the push–pull nature of both systems, indicating that the HOMO is predominantly localized on the donor fragment (D) and the first *π*-bridge (${\pi}_{1}$), representing 85.46% HOMO in QX-DMA and 87.40% in QX-TPA. However, the LUMO distribution reveals the greater effectiveness of the TPA group in promoting spatial charge separation. In the LUMO of QX-TPA, the electron density contribution on the donor fragment is drastically reduced to only 2.40%, being strongly shifted toward the acceptor units and adjacent bridges (A1, 42.73%; *π*_2_, 22.11%; A2, 24.95%). In contrast, the donor fragment of QX-DMA retains a significant 28.11% electron density on the LUMO. These results suggest that QX-TPA promotes a more efficient directional electron flow (D → A). This characteristic increases the physical separation between the injected electron and the hole, favoring electron injection processes and minimizing charge recombination at the dye-semiconductor interface.

### Photovoltaic properties

The calculated photovoltaic parameters discussed below were computed exclusively from molecular descriptors obtained at the DFT/TDDFT level. Therefore, the predicted *PCE* values should be interpreted as relative theoretical indicators intended for comparative purposes rather than absolute device efficiencies, since several experimental factors influencing DSSC performance are not explicitly considered in the present model [72].

The theoretical photovoltaic properties computed for both dyes are summarized in Table 3. QX-TPA exhibits a higher PCE value (1.85%) compared to QX-DMA (0.95%), suggesting for the former a greater DSSC efficiency.

**Table 3:** Computed photovoltaic parameters that influence dye efficiency

Dye	${J}_{SC}$(mA/cm^2^)	${V}_{OC}$(eV)	*LHE*	FF	*PCE (%)*
QX-DMA	1.042	1.03	0.916	0.885	0.95
QX-TPA	2.120	0.99	0.886	0.881	1.85

This trend is mainly associated with the higher computed short-circuit current density (${J}_{SC}$, Eq. (3)) of QX-TPA, which increases from 1.042 mA/cm^2^ (QX-DMA) to 2.120 mA/cm^2^ (QX-TPA). This enhanced ${J}_{SC}$ value indicates the existence of more efficient photoinduced charge-transfer processes, which may be related to improved excited-state charge separation and electron injection characteristics. Interestingly, although QX-DMA exhibits a slightly higher light harvesting efficiency (LHE, Eq. (4)) value (0.916) than QX-TPA (0.886), QX-TPA still exhibits superior predicted photovoltaic performance. This behavior suggests that parameters beyond photon absorption, such as excited-state stabilization, electron injection efficiency, and the suppression of charge recombination, also play important roles in determining the overall photovoltaic response.

In contrast, both sensitizers exhibit very similar open-circuit voltage (${V}_{OC}$, Eq. (2)) and fill factor (FF, Eq. (5)) values, with QX-DMA presenting slightly higher ${V}_{OC}$ (1.03 eV) and FF (0.885) values than QX-TPA (${V}_{OC}=0.99$ eV and FF=0.881). These relatively small differences indicate that the enhanced predicted photovoltaic performance of QX-TPA primarily originates from its higher short-circuit current density, rather than from voltage or charge-collection effects.

Although the calculated PCE values are lower than the experimentally reported efficiencies, the theoretical results successfully reproduce the experimental trend, with QX-TPA exhibiting higher ${J}_{SC}$ and PCE values than QX-DMA. As reported before [68], QX-TPA shows better photovoltaic performance (${J}_{SC}=18.37 \mathrm{m}\mathrm{A}/{\mathrm{c}\mathrm{m}}^{-2}$, ${V}_{OC}= 0.62 V$*,* $FF = 0.62 PCE=6.80\mathrm{\%})$ compared to QX-DMA (${J}_{SC}=15.91\mathrm{m}\mathrm{A}/{cm}^{-2}$, ${V}_{OC}= 0.57 V$*,* FF=0.59 and $PCE =5.34\mathrm{\%})$ thereby confirming the qualitative agreement between theory and experiment.

The influence of the arylamine donor groups DMA and TPA [94] on the thermodynamic and photochemical properties of the QX-DMA and QX-TPA dyes was examined using the computed DFT energetic parameters (Table 4). Both dyes have a D-*π*-A-*π*-A molecular architecture and exhibit efficient intramolecular charge-transfer pathways, although significant differences arise due to the electronic and structural features of the donor groups.

**Table 4:** Computed thermodynamic and energetic parameters for both dyes in eV

Dyes	${E}_{0-0}$	${E}_{dye}$	${E}_{dye}^{*}$	$\Delta {G}_{inject}$	$\Delta {G}_{reg}$	$\Delta {G}_{recomb}$
QX-DMA	1.70	4.97	3.18	7.18	− 9.15	0.97
QX-TPA	1.80	5.06	3.24	7.24	− 9.24	1.06

As shown in Table 4, QX-TPA displays a slightly higher ${E}_{0-0}$ value (1.80 eV) and a higher ground-state oxidation potential ${(E}_{dye}= 5.06 \mathrm{e}\mathrm{V})$ compared to QX-DMA (1.70 eV and 4.97 eV, respectively). Consequently, QX-TPA has a higher excited-state oxidation potential ${(E}_{dye}^{ *} = 3.24 \mathrm{e}\mathrm{V})$, indicating a stronger thermodynamic driving force for electron injection into the ${TiO}_{2}$ conduction band.

This thermodynamic analysis indicates that both dyes display highly favorable $\Delta {G}_{inject}$ values (7.18 and 7.24 eV for QX-DMA and QX-TPA, respectively), confirming that there is a sufficient driving force for electron injection from the photoexcited dye into the TiO2 conduction band. The slightly higher value of the TPA-based dye suggests a slightly enhanced thermodynamic driving force for charge separation upon photoexcitation.

Similarly, $\Delta {G}_{reg}$ values (-9.15 eV for QX-DMA and $-9.24 \mathrm{e}\mathrm{V}$ for QX-TPA) indicate that regeneration of the oxidized dye by the electrolyte is thermodynamically favorable in both cases. The more negative QX-TPA value suggests a more efficient regeneration process, which may be attributed to the stabilization of the oxidized HOMO level [114].

However, the calculated $\Delta {G}_{recomb}$ values (Table 4) reveal an opposite trend. QX-TPA exhibits a larger recombination driving force (1.06 eV) than QX-DMA (0.97 eV), indicating a less favorable back-electron transfer process and, consequently, a lower tendency toward charge recombination. These results suggest that while QX-DMA promotes a stronger excited-state charge separation through its larger dipole moment variation (see below), QX-TPA has a higher thermodynamic driving force for interfacial recombination. Therefore, the charge recombination behavior of these dyes is governed not only by the magnitude of charge displacement upon excitation but also by the energetic alignment of the oxidized dye state relative to the ${TiO}_{2}$ conduction band [119].

According to the data presented in Table 5, both dyes exhibit a positive change in dipole moment upon excitation, indicating a directional intramolecular charge transfer from the donor moiety toward the anchoring acceptor group. QX-DMA displays a larger dipole moment variation $\left(\Delta \mu = 3.23 D\right)$, with ${\mu}_{{S}_{0}} =14.0D$ and ${\mu}_{S1} =17.23 D$) than QX-TPA ($\Delta \mu = 2.21 D$, with ${\mu}_{{S}_{0}} =11.31 D$ and ${\mu}_{S1} = 13.2 D$). This larger variation indicates a more pronounced charge displacement in the excited state and a greater spatial separation between the injected electron and the oxidized dye for the DMA-functionalized system.

**Table 5:** DFT/TDDFT ground-state (${\mu}_{{S}_{0}}$) and excited-state (${\mu}_{S1}$) dipole moment magnitudes and corresponding dipole moment variation ($\Delta \mu$) in Debye (D) for QX-DMA and QX-TPA

Dyes	${\mu}_{{S}_{0}}$	${\mu}_{S1}$	$\Delta \mu$
QX-DMA	14.00	17.23	3.23
QX-TPA	11.31	13.52	2.21

An integrated analysis that combines thermodynamic parameters with kinetic descriptors, such as excited-state lifetimes and reorganization energies, is essential for predicting the photovoltaic performance of DSSCs [113]. This analysis is carried out in the next section.

### Excited-state lifetime and reorganization energies

Although both QX-DMA and QX-TPA dyes can inject electrons into the conduction band of the semiconductor, they have significant differences in their charge-transfer kinetics and overall efficiency. The excited-state lifetimes were computed from the TDDFT excitation energies and oscillator strengths of the first allowed electronic transition (${S}_{0} \to {S}_{1}$). The ${S}_{1}\to {S}_{0}$ fluorescence transition was selected because it corresponds to the lowest-energy singlet excited state and has the highest oscillator strength (f), in agreement with Kasha’s rule [99]. The evaluation of reorganization energies and excited-state lifetimes (Table 6) provides complementary supporting information to the previously discussed energetic descriptors, as shown in Table 6.

**Table 6:** Reorganization energy (λ) in eV and excited-state lifetime (τ) in ns

Dyes	${\lambda}_{h}$	${\lambda}_{e}$	${\lambda}_{t}$	τ(ns)
QX-DMA	1.65	3.67	5.33	0.437
QX-TPA	4.75	5.10	9.83	0.487

According to Marcus theory, the electron transfer rate decreases exponentially with increasing donor–acceptor distance. Consequently, the enhanced spatial separation in QX-TPA (see “Charge transfer analysis” Section) tends to reduce the recombination probability between the injected electron and the oxidized species, thereby extending the lifetime of the photogenerated charges. The enhanced spatial separation is compatible with the calculated excited-state lifetimes (τ) presented in Table 6: QX-TPA has a longer lifetime (0.487 ns) compared to QX-DMA (0.437 ns), suggesting that the TPA moiety improves the charge stabilization. Furthermore, the total reorganization energy (${\lambda}_{t}$) is significantly higher for QX-TPA (9.83 eV) than for QX-DMA (5.33 eV), driven by substantial contributions from both hole (${\lambda}_{h}= 4.75 \mathrm{e}\mathrm{V}$) and electron (${\lambda}_{e}= 5.10 \mathrm{e}\mathrm{V}$) components. This higher reorganization of energy can introduce an additional kinetic barrier to the back-electron transfer, further mitigating the undesirable interfacial charge recombination.

Although lower reorganization energies generally favor charge-transfer kinetics, the reorganization of energy is not an explicit component in the expression for short-circuit current density (${J}_{sc}$). According to the photocurrent model, ${J}_{sc}$ depends on the combined contributions of the light-harvesting efficiency (LHE), electron injection efficiency, dye regeneration efficiency and charge collection efficiency, as presented in Eq. (3) of the “Optoelectronic properties” Section. In the systems investigated in this work, both LHE and reorganization energies favor QX-DMA. However, the experimental results reported by Al-Marhabi et al. [68] show higher $({J}_{SC})$ and *PCE* values for QX-TPA. This apparent divergence indicates that light absorption and intrinsic charge-transport kinetics are not the sole factors governing the photovoltaic performance of this dye series. As discussed below, the intramolecular charge-transfer analysis obtained via TDDFT provides an explanation for this behavior. The q(CT) values calculated with CAM-B3LYP and ω B97X predict a more pronounced charge-transfer character for QX-DMA, which disagrees with the observed experimental trend. In contrast, the B3LYP functional correctly reproduces the experimental trend by predicting a greater charge-transfer character for QX-TPA. Furthermore, the fragment-based orbital composition, electron–hole separation, and natural transition orbital (NTO) analyses obtained with B3LYP indicate more efficient charge separation and a lower probability of recombination for QX-TPA.

### Reactivity descriptors

Efficient organic photosensitizers should exhibit lower IP values, favoring dye oxidation and the subsequent electron injection into the conduction band of ${TiO}_{2}$, and higher EA values, which facilitate electron acceptance and regeneration of the dye ground state [120, 121]. The computed chemical reactivity descriptors electrophilicity index (ω), the adiabatic ionization potential (IP), the electron affinity (EA), the chemical hardness (η), the electron-donating power (${\omega }^{-}$), the electron-accepting power (${\omega }^{+}$), the chemical potential (μ) and the electronegativity (χ) are presented in Table 7.

**Table 7:** Computed global hardness, chemical potential, electronegativity and electrophilicity index of the two dyes in eV

Dyes	IP	EA	η	χ	μ	ω	${\omega }^{+}$	${\omega }^{-}$
QX-DMA	5.81	2.06	1.87	3.93	− 3.93	4.13	2.40	6.33
QX-TPA	5.18	2.89	1.14	4.03	− 4.03	7.11	5.24	9.28

According to the IP and EA values, the QX-TPA dye has an appreciably lower ionization potential (5.18 eV) compared to QX-DMA (5.81 eV), indicating a lower energy barrier for dye oxidation and regeneration. In addition, QX-TPA shows a significantly higher electron affinity (2.89 eV) than QX-DMA (2.06 eV), indicating a greater ability to accept electrons and, consequently, a more efficient regeneration process.

Chemical hardness (η) is the resistance of a molecular system to changes in electron density under an external perturbation [122]. QX-DMA exhibits a higher hardness value (1.87 eV) than QX-TPA (1.14 eV), indicating that it is electronically harder. In contrast, the lower η QX-TPA value indicates greater polarizability and electronic reactivity (i.e., greater softness), thereby favoring intramolecular charge transfer.

Electronegativity (χ) reflects the molecule’s overall ability to attract electronic density [123]. The slightly higher χ value observed for QX-TPA (4.03 eV) compared to QX-DMA (3.93 eV) suggests greater efficiency of the former dye in stabilizing the redistributed charge after electronic excitation.

The tendency of an electron to escape from a system in its ground state is quantified by the chemical potential (μ) [124]. As shown in Table 5, the QX-TPA dye exhibits a slightly more negative chemical potential (− 4.03 eV) compared to QX-DMA (− 3.93 eV). This indicates that electrons in the QX-TPA dye have a lower probability of escaping from the system under equilibrium conditions, reflecting greater stabilization of the electronic density in the ground state.

The ability to accept electronic density is quantified by the global electrophilicity index ω. QX-TPA shows a significantly higher ω value (7.11 eV) compared to QX-DMA (4.13 eV), indicating greatly enhanced electronic reactivity. This behavior is further supported by the higher values of the electron-accepting power (${\omega }^{+},5.24 \mathrm{e}\mathrm{V}$) and the electron-donating power (${\omega }^{-} = 9.28 eV$) of QX-TPA compared to QX-DMA (${\omega }^{+}=2.40 \mathrm{e}\mathrm{V}$ and ${\omega }^{-}= 6.33 \mathrm{e}\mathrm{V}$), which confirms the greater electronic flexibility and charge-redistribution capability of the triphenylamine-based system.

In summary, global chemical reactivity descriptors indicate that the QX-TPA dye has higher electronic reactivity than QX-DMA. This behavior is relevant to dye-sensitized solar cells because it favors charge transfer and redistribution processes following photoexcitation.

### Charge-transfer analysis

**Fig. 4:**
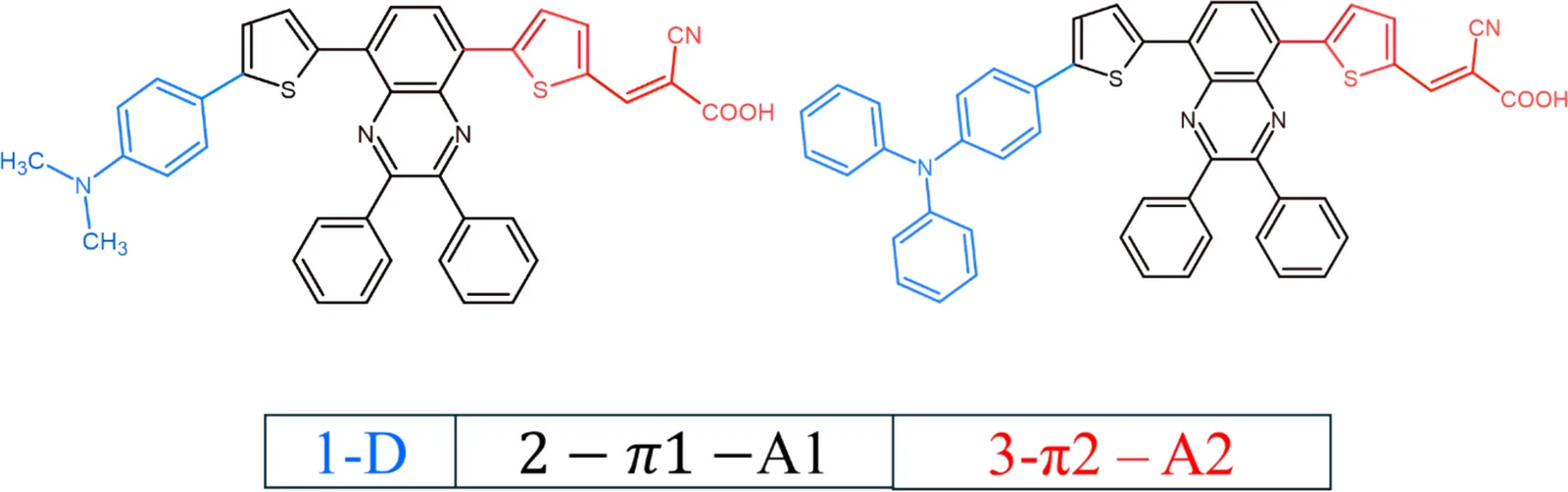
Fragmentation scheme used for the charge-transfer analysis of the QX-DMA and QX-TPA dyes based on the D-*π*-A-*π*-A architecture: donor unit (D, blue), first *π*-bridge and acceptor (π1-A1), in black, and second *π*-bridge with acceptor/anchoring group (π2-A2), in red

Now we examine charge-transfer effects, based on B3LYP/6-311G(d,p) TDDFT calculations. For this purpose, the molecular framework of both dyes was partitioned into three fragments according to the TheoDORE fragment-based charge-transfer protocol, in which the molecular fragments are explicitly defined as part of the input for the calculation of charge-transfer numbers and related electron–hole descriptors. As depicted in Fig. 4, the fragmentation scheme consists of (i) Fragment 1 (D), corresponding to the donor unit composed of the arylamine group (blue); (ii) Fragment 2 (π1-A1), consisting of the first thiophene unit acting as the π1-conjugated bridge and the 2,3-diphenylquinoxaline moiety as the first electron-acceptor unit (A1), in black; and (iii) Fragment 3 **(**π2-A2**)**, consisting of the second thiophene unit acting as the π2-conjugated bridge and the cyanoacrylic acid moiety as the second electron-acceptor and anchoring group (π2), in red.

**Fig. 5:**
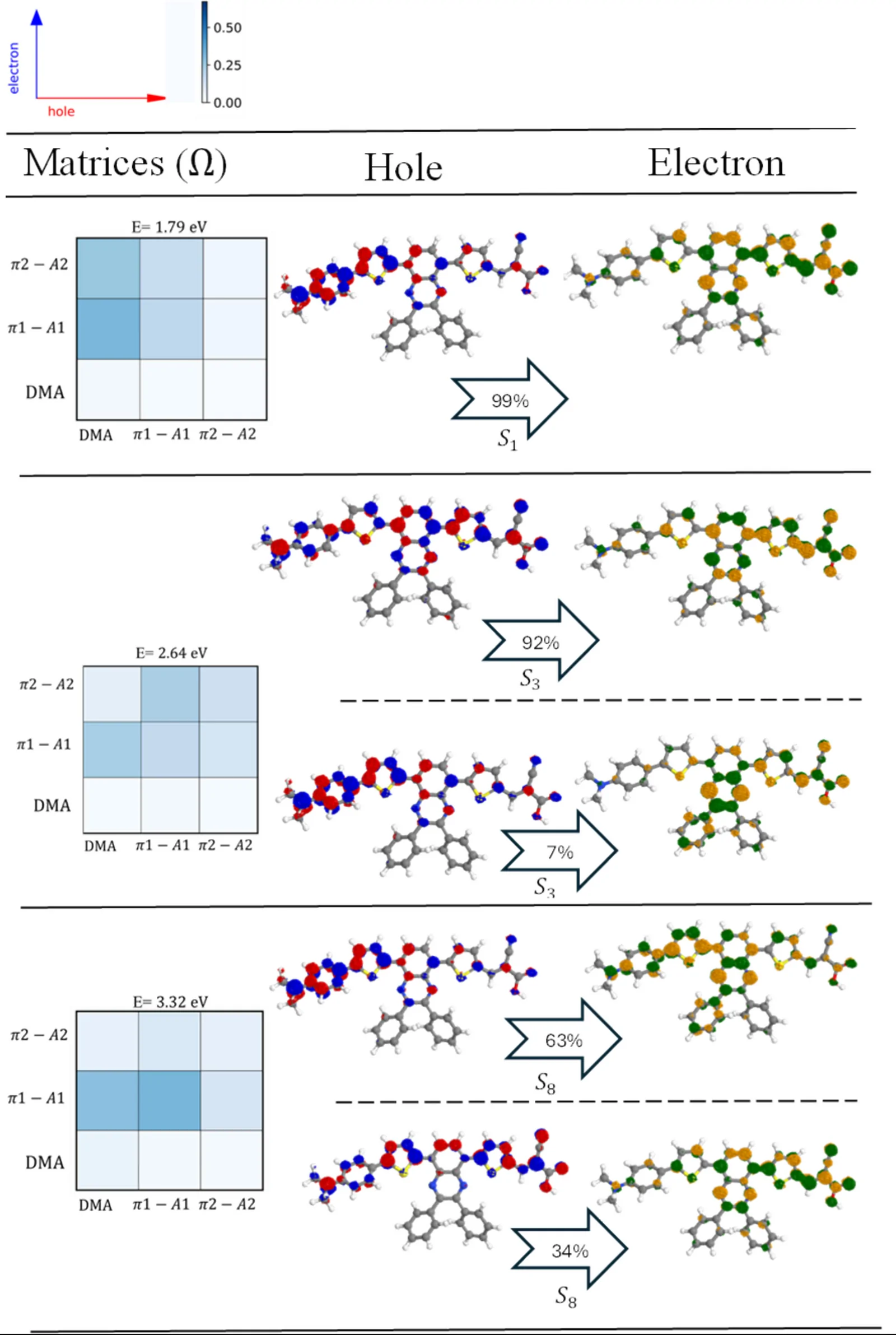
QX-DMA two-dimensional representation of the Ω matrix, which describes the electron–hole separation, together with the dominant NTOs and the corresponding excitation energies for the states ${S}_{1},{S}_{3}$, and ${S}_{8}$ transitions

**Fig. 6:**
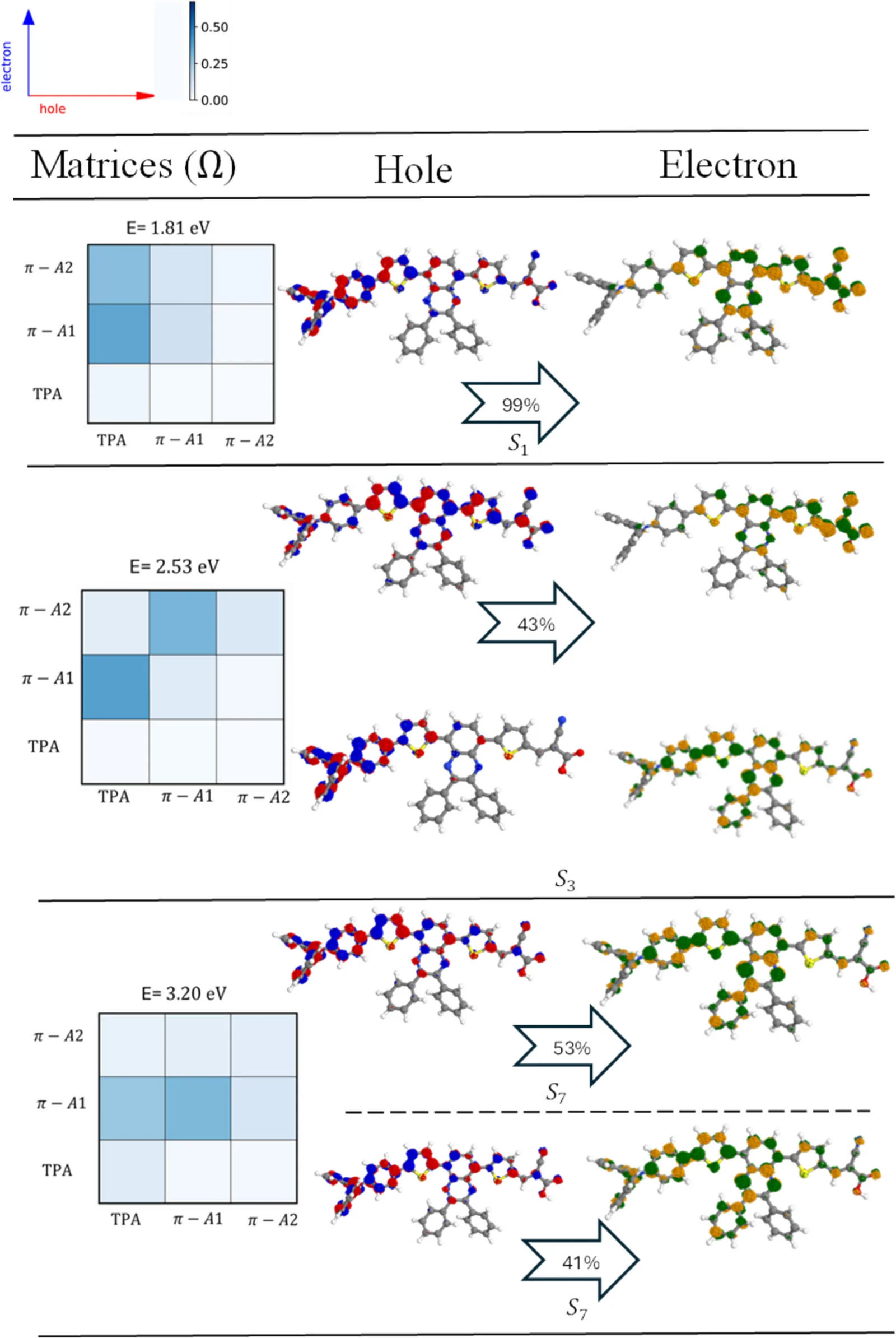
QX-TPA two-dimensional representation of the Ω matrix, which describes the electron–hole separation, and the dominant NTOs and the corresponding excitation energies for the ${S}_{1}, {S}_{3}, and\, {S}_{7}$ transitions

Figures 5 and 6 present the most relevant natural transition orbitals (NTOs) and the weights of the dominant NTO pair for the electronic excited states with the highest oscillator strengths, namely ${S}_{1}$,${S}_{3}$ and ${S}_{8}$ for QX-DMA, and ${S}_{1}$,${S}_{3}$ and ${S}_{7}$ for QX-TPA; the corresponding Ω matrix maps are also shown. In these maps, the horizontal axis represents the hole position and the vertical axis represents the electron position, enabling the characterization of the nature of the excitation: contributions along the diagonal indicate a localized excitation (LE) character, in which the hole and the electron remain within the same fragment, whereas off-diagonal terms reflect charge-transfer processes, evidencing the spatial separation between the electron and the hole across distinct fragments. The remaining NTOs and Ω matrix maps for QX-DMA and QX-TPA, corresponding to the other excited states, are provided in the Supporting Information (Figs. S2 and S3 for QX-DMA and Figs. S4 and S5 for QX-TPA).

The oscillator (f) strengths, charge-transfer indices q(CT), participation numbers (${PR}_{NTO}$), hole–electron separation distances ($\widetilde{{d}_{ex}}$), and wavelengths for QX-DMA are summarized in Table S6, while the data for QX-TPA are presented in Table S7 of the Supporting Information.

The two-dimensional maps represent, for each singlet transition (${S}_{1}-{S}_{10}$), the correlation between donor and acceptor regions of electronic density. Each (i,j) map element corresponds to a hole–electron transition between specific molecular fragments. For the lowest-energy states (transitions), particularly ${S}_{1}$, a pronounced separation between the hole and electron distributions was found, indicating a dominant charge-transfer character. For QX-DMA, the ${S}_{1}$ state exhibits a charge-transfer index $q\left(CT\right)=0.767e$ and a hole–electron separation $\widetilde{{d}_{ex} } = 10.51 {\overset \circ {\mathrm{A}} }$, indicating efficient electronic migration from the DMA donor toward the $\pi 1-\mathrm{A}1$ and $\pi 2-\mathrm{A}2$ acceptor fragments. Similarly, for QX-TPA, ${S}_{1}$ displays an even more pronounced CT character, with $q\left(CT\right)=0.805e$ and a significantly larger hole–electron separation ($\widetilde{{d}_{ex} } = 11.57 {\overset \circ {\mathrm{A}} }$), reflecting the greater extension and delocalization capability of the TPA donor. These results confirm that both dyes exhibit strong intramolecular charge-transfer character in the ${S}_{1}$ state, with a more intense effect observed for QX-TPA. The ${S}_{1}$ state is dominated by a HOMO → LUMO transition with a predominant intramolecular charge-transfer character, in which the hole remains localized on the donor group, while the electron density is transferred to the acceptor fragments. The ${PR}_{NTO} =1$ value indicates that this excitation is essentially governed by a single NTO pair.

The QX-DMA ${S}_{2}$ (Fig. S2, Supporting Information) and ${S}_{3}$ states exhibit high charge-transfer values, with q(CT)=0.729e and 0.673e respectively, associated with large hole–electron separations ($\widetilde{{d}_{ex} } =10.891 {\overset \circ {\mathrm{A}} }$ for ${S}_{2}$ and $\widetilde{{d}_{ex} } =8.295 {\overset \circ {\mathrm{A}} }$ for ${S}_{3}$). These results indicate that charge-transfer processes remain relevant even in higher-energy excited states. Other states, such as ${S}_{5}$ and ${S}_{6}$, also display high q(CT) values (0.606e and 0.743e, respectively), followed by significant hole–electron separations ($\widetilde{{d}_{ex} }=8.177 {\overset \circ {\mathrm{A}} }$ for ${S}_{5}$ and 9.800 ${\overset \circ {\mathrm{A}} }$ for ${S}_{6}$), evidencing charge-transfer processes in these states. The ${S}_{8}$ state is characterized by charge transfer from the DMA donor group to the π1-A1 fragment, with $q\left(CT\right)=0.577e$ and a hole–electron separation of $\widetilde{{d}_{ex} } =7.686 {\overset \circ {\mathrm{A}} }$, indicating that the electron remains predominantly localized on this fragment. Similarly, the ${S}_{9}$ state exhibits charge transfer from DMA to the π1-A1 fragment, followed by a secondary transfer between the π1-A1 and π2-A2 fragments, with q(CT)=0.598e and a hole–electron separation of $\widetilde{{d}_{ex} }=7.266 {\overset \circ {\mathrm{A}} }$. In contrast, the ${S}_{7}$ and ${S}_{10}$ states display lower q(CT) values (0.310e and 0.450e, respectively) and shorter hole–electron separations ($\widetilde{{d}_{ex} }=5.139 {\overset \circ {\mathrm{A}} }$ for ${S}_{7}$ and $\widetilde{{d}_{ex} }=7.538 {\overset \circ {\mathrm{A}} }$ for ${S}_{10}$), indicating that the hole and electron remain predominantly localized on the same molecular fragment, characterizing more localized excitations.

Similarly, for QX-TPA, the ${S}_{2}$ and ${S}_{3}$ states maintain a pronounced charge-transfer character, with q(CT) values ranging from 0.657e to 0.810e and hole–electron separations greater than $\widetilde{{d}_{ex} }=9.7 {\overset \circ {\mathrm{A}} }$, with the ${S}_{2}$ state exhibiting the largest spatial separation ($\widetilde{{d}_{ex} }=11.840 {\overset \circ {\mathrm{A}} }$). In addition, the ${S}_{5}$ and ${S}_{9}$ states also display high q(CT) values (>0.66e) and significant hole–electron separations, indicating that charge transfer extends over several excited states in QX-TPA. In contrast, excited states with lower q(CT) values (< 0.45*e*), such as ${S}_{8}$ and ${S}_{10}$, exhibit reduced hole–electron separations ($\widetilde{{d}_{ex} } < 6 {\overset \circ {\mathrm{A}} }$), reflecting more localized excitations. Overall, QX-TPA presents systematically higher q(CT) values and larger hole–electron separations across the series of excited states, indicating a more efficient and spatially more extensive charge separation compared to QX-DMA.

**Fig. 7:**
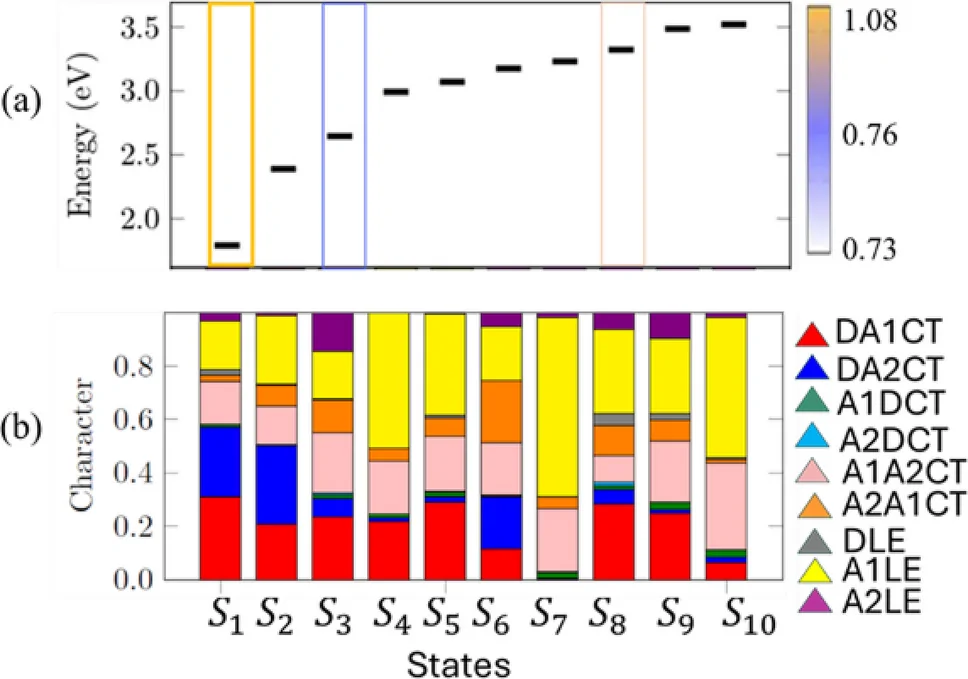
QX-DMA **a** excitation energies for the and oscillator strengths for the ${S}_{1}$, ${S}_{3}$, and ${S}_{8}$ states and **b** fragment-based analysis of the excited states ${S}_{1}-{S}_{10}$. Contributions of charge transfer (CT) and Local Excitation (LE) between the fragments DMA (D), 2,3-diphenylquinoxaline (A1), and cyanoacrylic acid (A2)

**Fig. 8:**
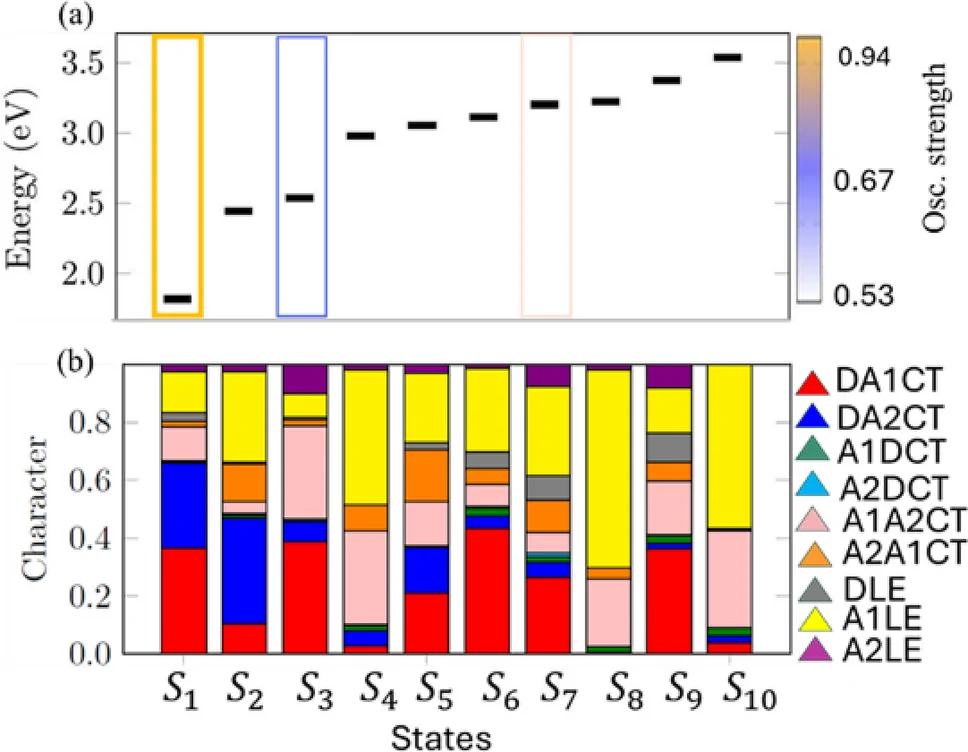
QX-TPA **a** excitation energies and oscillator strengths for the ${S}_{1}$, ${S}_{3}$, and ${S}_{7}$ states and **b** Fragment-based analysis of the excited states ${S}_{1}-{S}_{10}$. contributions of charge transfer ($q(\mathrm{C}\mathrm{T}$) and local excitation (LE) between the fragments TPA (D), 2,3-diphenylquinoxaline (A1), and cyanoacrylic acid (A2)

Figures 7 and 8, for each state, display excitation energies, oscillator strengths and charge transfer contributions. In Figs. 7a and 8a, the excitation energies of the first ten singlet states are shown, and their oscillator strengths are represented by color shading. This representation indicates that the ${S}_{1}$ bright state in QX-DMA has oscillator strength of 1.08, and QX-TPA has 0.94, located just below 2.0 eV. In addition, higher-energy bright states we found, namely ${S}_{3}$, and ${S}_{8}$, for QX-DMA, and ${S}_{3}$, and ${S}_{7}$, for QX-TPA.

In Figs. 7b (QX-DMA) and 8b (QX-TPA), bar charts decompose CT contributions for each excited state according to the three fragments defined in Fig. 4. Each bar indicates the relative contribution of the different types of electronic transitions according to the following labels: $\mathrm{D}\mathrm{A}1\mathrm{C}\mathrm{T}$ and $\mathrm{D}\mathrm{A}2\mathrm{C}\mathrm{T}$ correspond to the charge transfer from the donor to acceptors ($\mathrm{A}1$ and $\mathrm{A}2$, respectively); $\mathrm{A}1\mathrm{D}\mathrm{C}\mathrm{T}$ and $\mathrm{A}2\mathrm{D}\mathrm{C}\mathrm{T}$ represent the charge transfer from the acceptors to the donor; $\mathrm{A}1\mathrm{A}2\mathrm{C}\mathrm{T}$ and $\mathrm{A}2\mathrm{A}1\mathrm{C}\mathrm{T}$ are charge transfer between the two acceptors, in addition to local excitations: on the donor ($\mathrm{D}\mathrm{L}\mathrm{E}$) and on the acceptors ($\mathrm{A}1\mathrm{L}\mathrm{E}$ and $\mathrm{A}2\mathrm{L}\mathrm{E}$).

In agreement with the NTO analysis and the results for both dyes, Figs. 7b and 8b show that the ${S}_{1}$ bright state is predominantly dominated by charge-transfer processes from the donor to the acceptor groups. In particular, $\mathrm{D}\mathrm{A}1\mathrm{C}\mathrm{T}$ contributions (shown in red) associated with the charge transfer from the donor to acceptor A1 (2,3-diphenylquinoxaline), and the $\mathrm{D}\mathrm{A}2\mathrm{C}\mathrm{T}$ contributions (shown in blue), corresponding to the charge transfer from the donor to acceptor $\mathrm{A}2$(cyanoacrylic acid), are the most prominent. For QX-TPA, the charge transfer between these fragments is more pronounced compared to that observed for QX-DMA.

Overall, for both dyes, $\mathrm{D}\mathrm{A}1\mathrm{C}\mathrm{T}$ contributions are present in almost all excited states**.** The exceptions are the ${S}_{7}$ state of QX-DMA and the ${S}_{8}$ state of QX-TPA, which are characterized by predominantly local excitations on the acceptor $\mathrm{A}1$($\mathrm{A}1\mathrm{L}\mathrm{E}$) together with significant contributions from inter-acceptor charge transfer ($\mathrm{A}1\mathrm{A}2\mathrm{C}\mathrm{T}$)**.** As the excitation energy increases (${S}_{4}-{S}_{10}$), a progressive decrease in the $\mathrm{D}\mathrm{A}2\mathrm{C}\mathrm{T}$ contributions is observed, followed by an enhancement of localized excitations on acceptor A1 ($\mathrm{A}1\mathrm{L}\mathrm{E}$) and a corresponding reduction in the charge-transfer character involving acceptor $\mathrm{A}2$.

In addition to the B3LYP analysis, the charge-transfer character was examined using the CAM-B3LYP and *ω*B97X functionals to assess how the fraction of Hartree–Fock exchange and the inclusion of long-range exchange affect the description of the excited states. The analysis of electronic descriptors and intramolecular charge transfer reveals consistent differences between the QX-DMA and QX-TPA dyes, which exhibit a strong dependence on the type of exchange–correlation functional (B3LYP, CAM-B3LYP, and ω B97X) [125]. In particular, B3LYP, with its 20% fraction of Hartree–Fock exchange [50], presents a distinct performance, exhibiting larger q(CT) and ${d}_{ex}$ values for QX-TPA, whereas CAM-B3LYP and *ω*B97X predict a comparatively stronger CT character for QX-DMA.

A comparison of the excited-state descriptors obtained with CAM-B3LYP and *ω*B97X reveals a significant functional dependence in the predicted charge-transfer character of the low-lying excited states. As reported in the Supporting Information (Tables S8–S11), CAM-B3LYP predicts a markedly stronger charge-transfer excitation for QX-DMA, with (q(CT)=0.90e) and $\widetilde{{d}_{ex}} = 12.7{\overset \circ {\mathrm{A}} }$ for the ${S}_{1}$ state (Table S8), whereas the corresponding ωB97X values decrease to 0.361 Å and 5.401 Å, respectively (Table S9). For QX-TPA, a similar reduction is observed, with (q(CT)) decreasing from 0.44e to 0.348e and ($\widetilde{{d}_{ex}}$). from 6.579 to 5.255 Å when moving from CAM-B3LYP (Table S10) to ω B97X (Table S11).

The lower $q\left(CT\right)$ and $\widetilde{{d}_{ex}}$ values predicted by *ω*B97X indicate a smaller separation of the electron–hole pair, which can be attributed to the larger fraction of long-range Hartree–Fock exchange incorporated in this functional. Nevertheless, both functionals consistently describe the lowest excited states as donor-to-acceptor charge-transfer excitations. Moreover, the relatively low ${PR}_{NTO}$ values reported in the Supporting Information (Tables S8–S11) indicate that these excitations are dominated by a small number of NTO pairs, supporting a well-defined excitation mechanism. Therefore, despite quantitative differences in the charge-transfer descriptors, CAM-B3LYP and *ω*B97X provide a consistent qualitative description of the photoinduced charge-separation process for both dyes.

The relatively low ${PR}_{NTO}$ values are characteristic of predominantly single-excitation states with limited configurational mixing. Moreover, the similar ${PR}_{NTO}$ ranges obtained with CAM-B3LYP and *ω*B97X suggest that the qualitative nature of the electronic excitations may be independent of the functional employed. The *Ω* matrices along with the most representative NTO pairs are shown in Figs. S6–S9 of the Supporting Information.

In contrast to the QX-TPA dye, the decomposition of the excitation character obtained with CAM-B3LYP for QX-DMA reveals a significantly higher charge-transfer in the low-lying energy region (Figs. S10–S11). The lowest-lying excited state ${S}_{1}$, centered around 2.5 eV in the CAM-B3LYP calculations, exhibits a predominantly CT nature, driven heavily by the donor-to-acceptor-2 transition ($D{A}_{2}CT$, blue) alongside a standard ($D{A}_{1}CT$) contribution (red). Localized excitations on the acceptors (${A}_{1}LE$ and ${A}_{2}LE$) play a minor role in ${S}_{1}$ compared to what was observed for QX-TPA under the same level of theory.

Higher energy states calculated with the CAM-B3LYP range-separated functional also showcase distinct pathways; states 4, 7, and 10 are highly characterized by inter-acceptor charge transfer (${A}_{1}{A}_{2}CT$, pink), whereas states 6 and 7 retain strong $D{A}_{1}CT$ behavior. Furthermore, the donor localized excitation DLE, gray, which was a prominent feature of state 8 in QX-TPA, is drastically suppressed in QX-DMA when described by CAM-B3LYP, appearing only as a negligible contribution in states 6, 8, and 10.

The excitation character decomposition analysis (TDDFT/ωB97X) for the QX-DMA and QX-TPA compounds can be found in Figs. S12–S13 of the Supporting Information. A notable feature common to both derivatives is the absence of low-energy states with a purely donor–acceptor charge-transfer character. Instead, these states are largely dominated by local excitations (LE), with a strong predominance in acceptor fragment 1 (*A1LE*), accompanied by minor charge-transfer contributions.

## Conclusions

Using DFT and TDDFT, we rationalized the photophysical behavior of two molecules for dye-sensitized solar cell (DSSC) applications. Both dyes exhibit a similar excitation pattern, characterized by a fundamental HOMO → LUMO transition predominantly characterized by donor-to-acceptor charge transfer (CT), whereas higher-lying excited states display CT and local excitation (LE) mixed nature.

Fragment-resolved transition density analysis highlighted the decisive role of the donor unit in modulating the magnitude and spatial extension of the charge transfer. The quinoxaline moiety effectively acts as an intermediate electron acceptor bridge (${A}_{1}$), mediating the initial localization of the photoexcited electron and facilitating its subsequent migration toward the cyanoacrylic acid anchoring group (${A}_{2}$).

Quantitative B3LYP/6-311G(d,p) charge-transfer descriptors confirmed that QX-TPA exhibits a superior photophysical profile compared to QX-DMA. The triphenylamine (TPA) donor promotes highly efficient electronic delocalization and broader spatial charge separation than the dimethylamine (DMA) group. Consequently, substituting DMA with TPA optimizes critical properties for DSSC operation, specifically by enhancing the electron injection driving force and mitigating charge recombination pathways.

Importantly, our study underscored the functional dependence of these ICT parameters. While long-range corrected functionals exhibited a more significant CT character for QX-DMA compared to QX-TPA, the B3LYP functional with 20% exact  HF exchange accurately captured the extended electronic coupling, perfectly corroborating experimental electrochemical impedance pectroscopy trends, in which QX-TPA displays greater ICT character.

Although computational models inherently carry approximations regarding power conversion efficiencies, our results reproduce qualitative experimental trends, which validates our computational protocol. This theoretical framework establishes a reliable predictive tool for the rational in silico design of novel organic dyes, enabling the optimization of time and material resources by directing synthetic laboratory efforts toward the most theoretically promising architectures.

## Supplementary Information

Below is the link to the electronic supplementary material.

ESM 1 (DOCX 8.22 MB)

## Data Availability

The data that supports the findings of this study are available in the Supporting Information of this article. Additional data are available in GitHub and Zenodo and can be accessed at https://doi.org/10.5281/zenodo.18343811.

## References

[1] Yolcan OO (2023) World energy outlook and state of renewable energy: 10-year evaluation. Innovation and green development 2:100070. 10.1016/j.igd.2023.100070

[2] Korir BK, Kibet JK, Ngari SM (2024) A review on the current status of dye-sensitized solar cells: toward sustainable energy. Energy Sci Eng 12:3188–3226. 10.1002/ese3.1815

[3] Nnabuife SG, Ugbeh-Johnson J, Okeke NE, Ogbonnaya C (2022) Present and projected developments in hydrogen production: a technological review⁎. Carbon Capture Sci Technol 3:100042. 10.1016/j.ccst.2022.100042

[4] Dadi M, Siwale W, Munalula F et al (2025) A comprehensive review of advances in bioenergy including emerging trends and future directions. Discover Energy 5:26. 10.1007/s43937-025-00095-3

[5] Rezaei S, Hormaza Mejia A, Wu Y et al (2025) Global warming impacts of the transition from fossil fuel conversion and infrastructure to hydrogen. Appl Energy 397:126363. 10.1016/j.apenergy.2025.126363

[6] O’Regan B, Grätzel M (1991) A low-cost, high-efficiency solar cell based on dye-sensitized colloidal TiO2 films. Nature 353:737–740. 10.1038/353737a0

[7] Ren Y, Zhang D, Suo J et al (2023) Hydroxamic acid pre-adsorption raises the efficiency of cosensitized solar cells. Nature 613:60–65. 10.1038/s41586-022-05460-z 36288749

[8] Vallejo W, Lerma M, Díaz-Uribe C (2025) Dye sensitized solar cells: meta-analysis of effect sensitizer-type on photovoltaic efficiency. Heliyon 11:e41092. 10.1016/j.heliyon.2024.e41092 PMC11719345 39801998

[9] Badawy SA, Abdel-Latif E, Elmorsy MR (2024) Tandem dye-sensitized solar cells achieve 12.89% efficiency using novel organic sensitizers. Sci Rep 14:26072. 10.1038/s41598-024-75959-0 PMC11525810 39478023

[10] Aftabuzzaman M, Zhou H, Masud, Akman E, Boruah BD, Al-Ahmed A, Kim HK (2026) Progress in highly efficient and stable dye-sensitized solar cells: state-of-the-art materials and device fabrication. Coord Chem Rev 563:218020. 10.1016/j.ccr.2026.218020

[11] Michaels H, Rinderle M, Freitag R et al (2020) Dye-sensitized solar cells under ambient light powering machine learning: towards autonomous smart sensors for the internet of things. Chem Sci 11:2895–2906. 10.1039/C9SC06145B PMC8157489 34122790

[12] Barichello J, Mariani P, Vesce L et al (2024) Bifacial dye-sensitized solar cells for indoor and outdoor renewable energy-based application. J Mater Chem C Mater 12:2317–2349. 10.1039/D3TC03220E

[13] Venkatesan S, Hsu T-H, Teng H, Lee Y-L (2023) Dye‐sensitized solar cells with efficiency over 36% under ambient light achieved by cosensitized tandem structure. Solar RRL 7:2300220. 10.1002/solr.202300220

[14] Qamar S, Erten Ela S (2024) Dye-sensitized solar cells (DSSC): principles, materials and working mechanism. Curr Opin Colloid Interface Sci 74:101871. 10.1016/j.cocis.2024.101871

[15] Sharma K, Sharma V, Sharma SS (2018) Dye-sensitized solar cells: fundamentals and current status. Nanoscale Res Lett 13:381. 10.1186/s11671-018-2760-6 PMC6261913 30488132

[16] Thomas S, Deepak TG, Anjusree GS et al (2014) A review on counter electrode materials in dye-sensitized solar cells. J Mater Chem A 2:4474–4490. 10.1039/C3TA13374E

[17] Mei J, Leung NLC, Kwok RTK et al (2015) Aggregation-induced emission: together we shine, united we soar! Chem Rev 115:11718–11940. 10.1021/acs.chemrev.5b00263 26492387

[18] Wang Y, Shi Y, Cong L, Li H (2015) TDDFT study of twisted intramolecular charge transfer and intermolecular double proton transfer in the excited state of 4′-dimethylaminoflavonol in ethanol solvent. Spectrochim Acta A Mol Biomol Spectrosc 137:913–918. 10.1016/j.saa.2014.09.024 25282020

[19] Masud KHK (2023) Redox shuttle-based electrolytes for dye-sensitized solar cells: comprehensive guidance, recent progress, and future perspective. ACS Omega 8:6139–6163. 10.1021/acsomega.2c06843 PMC9948191 36844550

[20] Biney J, Madou M, Jabbour G, Park J (2025) 2D composite materials for electrodes in dye-sensitized solar cells─an overview. ACS Appl Mater Interfaces 17:17855–17880. 10.1021/acsami.4c13963 40096654

[21] Yadagiri B, Kumar Kaliamurthy A, Yoo K et al (2023) Molecular engineering of photosensitizers for solid-state dye-sensitized solar cells: recent developments and perspectives. ChemOpen 12:e202300170. 10.1002/open.202300170 PMC10695739 37874016

[22] Wooh S, Kim T-Y, Song D et al (2015) Surface modification of TiO_2_ photoanodes with fluorinated self-assembled monolayers for highly efficient dye-sensitized solar cells. ACS Appl Mater Interfaces 7:25741–25747. 10.1021/acsami.5b07211 26506252

[23] Malhotra SS, Ahmed M, Gupta MK, Ansari A (2024) Metal-free and natural dye-sensitized solar cells: recent advancements and future perspectives. Sustain Energy Fuels 8:4127–4163. 10.1039/D4SE00406J

[24] Yahya M, Bouziani A, Ocak C et al (2021) Organic/metal-organic photosensitizers for dye-sensitized solar cells (DSSC): recent developments, new trends, and future perceptions. Dyes Pigments 192:109227. 10.1016/j.dyepig.2021.109227

[25] Rattanawan R, Promarak V, Sudyoadsuk T et al (2016) Theoretical design of coumarin derivatives incorporating auxiliary acceptor with D-*π*-A-*π*-A configuration for dye-sensitized solar cells. J Photochem Photobiol A Chem 322–323:16–26. 10.1016/j.jphotochem.2016.02.016

[26] Kacimi R, Raftani M, Abram T et al (2021) Theoretical design of D-*π*-A system new dyes candidate for DSSC application. Heliyon 7:e07171. 10.1016/j.heliyon.2021.e07171 PMC8214095 34179523

[27] Yang L, Wu Y, Murugan P et al (2024) Impact of different *π*-bridges on the photovoltaic performance of A-D-D′-D-A small molecule-based donors. Molecules 29:4231. 10.3390/molecules29174231 PMC11396980 39275079

[28] Yang C, Song P, El-Shishtawy RM et al (2021) Photovoltaic performance and power conversion efficiency prediction of double fence porphyrins. Phys Chem Chem Phys 23:27042–27058. 10.1039/D1CP03593B 34847208

[29] Ye C, Cheng H, Wrede S et al (2023) Charge recombination deceleration by lateral transfer of electrons in dye-sensitized NiO photocathode. J Am Chem Soc 145:11067–11073. 10.1021/jacs.3c00269 PMC10214442 37191461

[30] Madrid-Úsuga D, Suárez OJ, Portacio A (2025) Impact of molecular *π*-bridge modifications on triphenylamine-based donor materials for organic photovoltaic solar cells. Condens Matter 10:52. 10.3390/condmat10040052

[31] Ueda M, Nagayama R, Nagaoka M et al (2025) Synthesis and electronic properties of novel donor–*π*–acceptor-type functional dyes with a carbonyl-bridged bithiophene *π*-spacer. Molecules 30:3084. 10.3390/molecules30153084 PMC12348573 40807260

[32] David Ndaleh DN, Nugegoda D, Watson J et al (2021) Donor group influence on dye-sensitized solar cell device performances: balancing dye loading and donor size. Dyes Pigments 187:109074. 10.1016/j.dyepig.2020.109074

[33] Roohi H, Mohtamadifar N (2022) The role of the donor group and electron-accepting substitutions inserted in *π*-linkers in tuning the optoelectronic properties of D–*π*–A dye-sensitized solar cells: a DFT/TDDFT study. RSC Adv 12:11557–11573. 10.1039/D2RA00906D PMC9006569 35425060

[34] Fan S, Lv K, Sun H et al (2015) The position effect of electron-deficient quinoxaline moiety in porphyrin based sensitizers. J Power Sources 279:36–47. 10.1016/j.jpowsour.2014.12.143

[35] Borges I, Aquino AJA, Köhn A et al (2013) Ab initio modeling of excitonic and charge-transfer states in organic semiconductors: the PTB1/PCBM low band gap system. J Am Chem Soc 135:18252–18255. 10.1021/ja4081925 24215627

[36] Cardozo TM, Aquino AJA, Barbatti M et al (2015) Absorption and fluorescence spectra of poly(*p*-phenylenevinylene) (PPV) oligomers: an ab initio simulation. J Phys Chem A 119:1787–1795. 10.1021/jp508512s PMC4353058 25415930

[37] Aquino AAJ, Borges I, Nieman R et al (2014) Intermolecular interactions and charge transfer transitions in aromatic hydrocarbon–tetracyanoethylene complexes. Phys Chem Chem Phys 16:20586–20597. 10.1039/C4CP02900C 25156236

[38] Borges I, Uhl E, Modesto-Costa L et al (2016) Insight into the excited state electronic and structural properties of the organic photovoltaic donor polymer poly(thieno[3,4-*b*]thiophene benzodithiophene) by means of ab initio and density functional theory. J Phys Chem C 120:21818–21826. 10.1021/acs.jpcc.6b07689

[39] Rosa NMP, Borges I (2025) Review on the DFT computation of bulk heterojunction and dye-sensitized organic solar cell properties. J Mol Model 31:83. 10.1007/s00894-025-06304-z 39945938

[40] Rosa NMP, Borges I (2024) Photophysical properties of donor (D)–acceptor (A)–donor (D) diketopyrrolopyrrole (A) systems as donors for applications to organic electronic devices. J Comput Chem 45:2885–2898. 10.1002/jcc.27492 39212065

[41] Monteiro-de-Castro G, Borges I (2023) A Hammett’s analysis of the substituent effect in functionalized diketopyrrolopyrrole (DPP) systems: optoelectronic properties and intramolecular charge transfer effects. J Comput Chem 44:2256–2273. 10.1002/jcc.27195 37496237

[42] Modesto-Costa L, Borges I, Aquino AJA, Lischka H (2018) Electronic structure theory gives insights into the higher efficiency of the PTB electron-donor polymers for organic photovoltaics in comparison with prototypical P3HT. J Chem Phys 149:184905. 10.1063/1.5054919 30441933

[43] Belbah H, Bashir M, Siddique F et al (2025) Charge transfer interactions and electronic structure insights of quercetin 3-O-rutinoside complexes with *π*-acceptors: a DFT and spectroscopic investigation. Mol Phys. 10.1080/00268976.2025.2579187

[44] Zhang L, Cole JM (2017) Dye aggregation in dye-sensitized solar cells. J Mater Chem A Mater 5:19541–19559. 10.1039/C7TA05632J

[45] Xu F, Testoff TT, Wang L, Zhou X (2020) Cause, regulation and utilization of dye aggregation in dye-sensitized solar cells. Molecules 25:4478. 10.3390/molecules25194478 PMC7582523 33003462

[46] Lazrak M, Toufik H, Bouzzine SM et al (2023) Theoretical analysis on D-*π*-A triphenylamine-based dyes for dye-sensitized solar cells: effect of *π*-bridges on the optoelectronic, and photovoltaic properties. J Mol Model 29:266. 10.1007/s00894-023-05660-y 37505323

[47] Neese F (2025) Software update: the ORCA program system—version 6.0. WIREs Comput Mol Sci 15:e70019. 10.1002/wcms.70019

[48] Krishnan R, Binkley JS, Seeger R, Pople JA (1980) Self-consistent molecular orbital methods. XX. A basis set for correlated wave functions. J Chem Phys 72:650–654. 10.1063/1.438955

[49] Perdew JP, Burke K, Ernzerhof M (1996) Generalized gradient approximation made simple. Phys Rev Lett 77:3865–3868. 10.1103/PhysRevLett.77.3865 10062328

[50] Becke AD (1993) Density-functional thermochemistry. III. The role of exact exchange. J Chem Phys 98:5648–5652. 10.1063/1.464913

[51] Grimme S, Ehrlich S, Goerigk L (2011) Effect of the damping function in dispersion corrected density functional theory. J Comput Chem 32:1456–1465. 10.1002/jcc.21759 21370243

[52] Hehre WJ, Ditchfield R, Pople JA (1972) Self—consistent molecular orbital methods. XII. Further extensions of gaussian—type basis sets for use in molecular orbital studies of organic molecules. J Chem Phys 56:2257–2261. 10.1063/1.1677527

[53] Hariharan PC, Pople JA (1973) The influence of polarization functions on molecular orbital hydrogenation energies. Theor Chim Acta 28:213–222. 10.1007/BF00533485

[54] Lu T, Chen F (2012) Multiwfn: a multifunctional wavefunction analyzer. J Comput Chem 33:580–592. 10.1002/jcc.22885 22162017

[55] Yanai T, Tew DP, Handy NC (2004) A new hybrid exchange–correlation functional using the Coulomb-attenuating method (CAM-B3LYP). Chem Phys Lett 393:51–57. 10.1016/j.cplett.2004.06.011

[56] Chai J-D, Head-Gordon M (2008) Systematic optimization of long-range corrected hybrid density functionals. J Chem Phys 128:084106. 10.1063/1.2834918 18315032

[57] Zhao Y, Truhlar DG (2008) The M06 suite of density functionals for main group thermochemistry, thermochemical kinetics, noncovalent interactions, excited states, and transition elements: two new functionals and systematic testing of four M06-class functionals and 12 other functionals. Theor Chem Acc 120:215–241. 10.1007/s00214-007-0310-x

[58] Stahn M, Ehlert S, Grimme S (2023) Extended conductor-like polarizable continuum solvation model (CPCM-X) for semiempirical methods. J Phys Chem A 127:7036–7043. 10.1021/acs.jpca.3c04382 37567769

[59] Cancès E, Mennucci B, Tomasi J (1997) A new integral equation formalism for the polarizable continuum model: theoretical background and applications to isotropic and anisotropic dielectrics. J Chem Phys 107:3032–3041. 10.1063/1.474659

[60] Franck J, Dymond EG (1926) Elementary processes of photochemical reactions. Trans Faraday Soc 21:536. 10.1039/tf9262100536

[61] Condon E (1926) A theory of intensity distribution in band systems. Phys Rev 28:1182–1201. 10.1103/PhysRev.28.1182

[62] Verhoeven JW (1996) Glossary of terms used in photochemistry (IUPAC recommendations 1996). Pure Appl Chem 68:2223–2286. 10.1351/pac199668122223

[63] Păușescu I, Todea A, Dreavă D-M et al (2022) Experimental and computational studies on bio-inspired flavylium salts as sensitizers for dye-sensitized solar cells. Materials 15:6985. 10.3390/ma15196985 PMC9572272 36234326

[64] Coetzee L-CC, Adeyinka AS, Magwa N (2022) A theoretical evaluation of the efficiencies of metal-free 1,3,4-oxadiazole dye-sensitized solar cells: insights from electron–hole separation distance analysis. Energies (Basel) 15:4913. 10.3390/en15134913

[65] Plasser F (2020) TheoDORE: a toolbox for a detailed and automated analysis of electronic excited state computations. J Chem Phys 152:084108. 10.1063/1.5143076 32113349

[66] Plasser F, Lischka H (2012) Analysis of excitonic and charge transfer interactions from quantum chemical calculations. J Chem Theory Comput 8:2777–2789. 10.1021/ct300307c 26592119

[67] MATLAB (R2025a), Natick, Massachusetts: The MathWorks Inc.; 2025.

[68] Al-Marhabi AR, El-Shishtawy RM, Al-Footy KO et al (2025) The impact of aryl amine donors on the performance of dye-sensitized solar cells based on quinoxaline D-*π*-A-*π*-A sensitizers. J Mol Struct 1333:141746. 10.1016/j.molstruc.2025.141746

[69] Nam S-H, Ju D-W, Boo J-H (2024) Variations in power conversion efficiency on n-type dye-sensitized solar cells with synthesized TiO2 nanoparticle: a thickness effect of active layer. Catalysts 14:598. 10.3390/catal14090598

[70] Al-Qurashi OS, Wazzan N (2021) Prediction of power conversion efficiencies of diphenylthienylamine-based dyes adsorbed on the titanium dioxide nanotube. ACS Omega 6:8967–8975. 10.1021/acsomega.0c06340 PMC8028126 33842767

[71] Xing F-L, Zhang Z-H, Yang C-L et al (2022) Phycoerythrobilin/phycourobilin as efficient sensitizers of dye-sensitized solar cell. Sol Energy 243:494–499. 10.1016/j.solener.2022.08.028

[72] Khadiri A, Warad I, Abuelizz HA et al (2024) Predicting photovoltaic parameters using DFT and TD-DFT calculations of novel triphenylamine-based organic dyes: the effect of the internal or auxiliary acceptors on photovoltaic performance for DSSC. Sol Energy 279:112832. 10.1016/j.solener.2024.112832

[73] Yang C, Liu T, Song P et al (2022) Revealing the photoelectric performance and multistep electron transfer mechanism in D-A-π-A dyes coupled with a chlorophyll derivative for co-sensitized solar cells. J Mol Liq 368:120797. 10.1016/j.molliq.2022.120797

[74] Lee M-H (2022) Identifying correlation between the open-circuit voltage and the frontier orbital energies of non-fullerene organic solar cells based on interpretable machine-learning approaches. Sol Energy 234:360–367. 10.1016/j.solener.2022.02.010

[75] Singh M, Kanaparthi RK (2022) Theoretical exploration of 1,3-Indanedione as electron acceptor-cum-anchoring group for designing sensitizers towards DSSC applications. Sol Energy 237:456–469. 10.1016/j.solener.2022.01.018

[76] Anthony WO, Abubakar MK, Obiyenwa KG et al (2025) Computational design of phenoxazine and phenothiazine dye sensitizers with improved charge separation for application in DSSCs: a DFT study. Discover Chemistry 2:263. 10.1007/s44371-025-00352-3

[77] Lu T-F, Li W, Bai F-Q et al (2017) Anionic ancillary ligands in cyclometalated Ru(II) complex sensitizers improve photovoltaic efficiency of dye-sensitized solar cells: insights from theoretical investigations. J Mater Chem A Mater 5:15567–15577. 10.1039/C7TA03360E

[78] Ding Y, Zhu C, Zhang J, Zhu H (2025) Computational molecular design of new 3D structured quinoxaline dyes with panchromatic NIR absorption and enhanced stability enabling efficient copper-based dye sensitized solar cells. J Fluoresc 35:13575–13593. 10.1007/s10895-025-04477-7 40938497

[79] Zhang J, Zhang J-Z, Li H-B et al (2014) Rational modifications on champion porphyrin dye SM315 using different electron-withdrawing moieties toward high performance dye-sensitized solar cells. Phys Chem Chem Phys 16:24994–25003. 10.1039/C4CP03355H 25327722

[80] Xu Y, Li M, Fu Y et al (2019) Theoretical study of high-efficiency organic dyes with the introduction of different auxiliary heterocyclic acceptors based on IQ1 toward dye-sensitized solar cells. J Mol Graph Model 86:170–178. 10.1016/j.jmgm.2018.10.001 30368087

[81] Fu Y, Lu T, Xu Y et al (2018) Theoretical screening and design of SM315-based porphyrin dyes for highly efficient dye-sensitized solar cells with near-IR light harvesting. Dyes Pigments 155:292–299. 10.1016/j.dyepig.2018.03.045

[82] Sung HK, Lee Y, Kim WH et al (2020) Enhanced power conversion efficiency of dye-sensitized solar cells by band edge shift of TiO2 photoanode. Molecules 25:1502. 10.3390/molecules25071502 PMC7181208 32224956

[83] Zheng L, Polizzi NF, Dave AR et al (2016) Where is the electronic oscillator strength? Mapping oscillator strength across molecular absorption spectra. J Phys Chem A 120:1933–1943. 10.1021/acs.jpca.6b00692 PMC4849861 26950828

[84] Conradie J (2024) Effective dyes for DSSCs–important experimental and calculated parameters. Energy Nexus 13:100282. 10.1016/j.nexus.2024.100282

[85] Lee M-H (2024) Predicting and analyzing the fill factor of non-fullerene organic solar cells based on material properties and interpretable machine-learning strategies. Sol Energy 267:112191. 10.1016/j.solener.2023.112191

[86] Preat J, Michaux C, Jacquemin D, Perpète EA (2009) Enhanced efficiency of organic dye-sensitized solar cells: triphenylamine derivatives. The Journal of Physical Chemistry C 113:16821–16833. 10.1021/jp904946a

[87] Al-Fatlawe BA, AL-Temimei FA (2025) Optimizing electronic and photovoltaic properties of D–*π*–A organic dyes for DSSCs using density functional theory. J Fluoresc 35:7833–7845. 10.1007/s10895-024-04118-5 39798021

[88] Wach A, Zou X, Wojtaszek K et al (2023) Towards understanding the TiO_2_ doping at the surface and bulk. X-Ray Spectrom 52:261–268. 10.1002/xrs.3363

[89] Gunawardhana P, Balasooriya Y, Kandanapitiye MS et al (2023) Optoelectronic characterization of natural dyes in the quest for enhanced performance in dye-sensitized solar cells: a density functional theory study. Appl Sci (Basel) 14:188. 10.3390/app14010188

[90] Dandrade B, Datta S, Forrest S et al (2005) Relationship between the ionization and oxidation potentials of molecular organic semiconductors. Org Electron 6:11–20. 10.1016/j.orgel.2005.01.002

[91] Samiee S, Mohammadi landi H (2024) The impact of carboxylic acid anchoring groups on the optoelectronic and nonlinear optical properties of Ru complex dyes: a computational approach. J Saudi Chem Soc 28:101793. 10.1016/j.jscs.2023.101793

[92] Daeneke T, Mozer AJ, Uemura Y et al (2012) Dye regeneration kinetics in dye-sensitized solar cells. J Am Chem Soc 134:16925–16928. 10.1021/ja3054578 23013038

[93] Zdyb A, Krawczak E (2021) Organic dyes in dye-sensitized solar cells featuring back reflector. Energies 14:5529. 10.3390/en14175529

[94] Azaid A, Raftani M, Alaqarbeh M et al (2022) New organic dye-sensitized solar cells based on the D-A–*π*–A structure for efficient DSSCs: DFT/TD-DFT investigations. RSC Adv 12:30626–30638. 10.1039/D2RA05297K PMC9597288 36337973

[95] Jacquemin D (2016) Excited-state dipole and quadrupole moments: TD-DFT versus CC2. J Chem Theory Comput 12:3993–4003. 10.1021/acs.jctc.6b00498 PMC4980690 27385324

[96] Sarkar R, Boggio-Pasqua M, Loos P-F, Jacquemin D (2021) Benchmarking TD-DFT and wave function methods for oscillator strengths and excited-state dipole moments. J Chem Theory Comput 17:1117–1132. 10.1021/acs.jctc.0c01228 33492950

[97] Marcus RA (1965) On the theory of electron-transfer reactions. VI. Unified treatment for homogeneous and electrode reactions. J Chem Phys 43:679–701. 10.1063/1.1696792

[98] López-Estrada O, Laguna HG, Barrueta-Flores C, Amador-Bedolla C (2018) Reassessment of the four-point approach to the electron-transfer Marcus–Hush theory. ACS Omega 3:2130–2140. 10.1021/acsomega.7b01425 PMC6641260 31458519

[99] Kasha M (1950) Characterization of electronic transitions in complex molecules. Discuss Faraday Soc 9:14. 10.1039/df9500900014

[100] Khadiri A, Thakur A, Warad I et al (2025) DFT and TD-DFT calculations to estimate the photovoltaic parameters of some metal-free dyes based on triphenylamine: the influence of inserting an auxiliary electron-withdrawing group on DSSC’s performance. RSC Adv 15:26807–26829. 10.1039/D5RA04785D PMC12302405 40727291

[101] Tommalieh MJ, Aljameel AI, Hussein RK et al (2024) The effect of conjugated nitrile structures as acceptor moieties on the photovoltaic properties of dye-sensitized solar cells: DFT and TD-DFT investigation. Int J Mol Sci 25:7138. 10.3390/ijms25137138 PMC11241837 39000245

[102] Cafiero M (2024) Role of exact exchange and empirical dispersion in density functional theory-based three-body noncovalent interactions. J Phys Chem A 128:8777–8786. 10.1021/acs.jpca.4c03262 PMC11472333 39320094

[103] Mester D, Kállay M (2023) Vertical ionization potentials and electron affinities at the double-hybrid density functional level. J Chem Theory Comput 19:3982–3995. 10.1021/acs.jctc.3c00363 PMC10339736 37326360

[104] Chattaraj PK, Sarkar U, Roy DR (2006) Electrophilicity index. Chem Rev 106:2065–2091. 10.1021/cr040109f 16771443

[105] Parr RG, Donnelly RA, Levy M, Palke WE (1978) Electronegativity: the density functional viewpoint. J Chem Phys 68:3801–3807. 10.1063/1.436185

[106] Pearson RG (2005) Chemical hardness and density functional theory. J Chem Sci 117:369–377. 10.1007/BF02708340

[107] Parr RG, Yang W (1995) Density-functional theory of the electronic structure of molecules. Annu Rev Phys Chem 46:701–728. 10.1146/annurev.pc.46.100195.003413 24341393

[108] Barrales-Martínez C, Durán R, Jaque P (2023) New insights into H_2_ activation by intramolecular frustrated Lewis pairs based on aminoboranes: the local electrophilicity index of boron as a suitable indicator to tune the reversibility of the process. Chem Sci 14:11798–11808. 10.1039/D3SC03992G PMC10619628 37920343

[109] Omidvar A, Fazeli F, Ghaed-Sharaf T, Keshavarzi R (2024) Fine structural tuning of diphenylaniline-based dyes for designing semiconductors relevant to dye-sensitized solar cells. Sci Rep 14:26231. 10.1038/s41598-024-77953-y PMC11528015 39482468

[110] Piontkowski Z, McCamant DW (2018) Excited-state planarization in donor–bridge dye sensitizers: phenylene versus thiophene bridges. J Am Chem Soc 140:11046–11057. 10.1021/jacs.8b05463 30091908

[111] Kim S-Y, Cho Y-J, Lee A-R et al (2017) Influence of *π*-conjugation structural changes on intramolecular charge transfer and photoinduced electron transfer in donor–*π*–acceptor dyads. Phys Chem Chem Phys 19:426–435. 10.1039/C6CP06566J 27905585

[112] Shakil JA, Saikat SP, Bhattacharjee N et al (2024) DFT/TD-DFT study of novel triphenylamine-based dyes with azo moieties and *π*-spacer variations for enhanced dye-sensitized solar cell performance. Chem Phys Impact 9:100725. 10.1016/j.chphi.2024.100725

[113] Zhang W, Jiang H, Yu M et al (2023) D-A-*π*-A organic dyes with fluorenyl-substituted bulky donor for efficient dye-sensitized solar cells. Green Chem Eng 4:393–398. 10.1016/j.gce.2022.07.005

[114] Chordiya K, Ali MdE, Kahaly MU (2022) Photoexcited intramolecular charge transfer in dye sensitizers: predictive *in silico* screening for dye-sensitized solar cell devices. ACS Omega 7:13465–13474. 10.1021/acsomega.1c06233 PMC9088764 35559159

[115] Kubo W, Sakamoto A, Kitamura T et al (2004) Dye-sensitized solar cells: improvement of spectral response by tandem structure. J Photochem Photobiol A Chem 164:33–39. 10.1016/j.jphotochem.2004.01.024

[116] Lee KE, Gomez MA, Elouatik S, Demopoulos GP (2010) Further understanding of the adsorption mechanism of N719 sensitizer on anatase TiO_2_ films for DSSC applications using vibrational spectroscopy and confocal Raman imaging. Langmuir 26:9575–9583. 10.1021/la100137u 20429522

[117] Li M, Kou L, Diao L et al (2015) Theoretical study of WS-9-based organic sensitizers for unusual Vis/NIR absorption and highly efficient dye-sensitized solar cells. J Phys Chem C 119:9782–9790. 10.1021/acs.jpcc.5b03667

[118] Zhong L, Wang X, Rong M (2018) Molecular orbital composition and its effect on electron-impact ionization cross sections of molecules: a comparative study. Phys Plasmas 25:103507. 10.1063/1.5053903

[119] Wiberg J, Marinado T, Hagberg DP et al (2010) Distance and driving force dependencies of electron injection and recombination dynamics in organic dye-sensitized solar cells. J Phys Chem B 114:14358–14363. 10.1021/jp1002963 20380364

[120] Shao Y, Mei Y, Sundholm D, Kaila VRI (2020) Benchmarking the performance of time-dependent density functional theory methods on biochromophores. J Chem Theory Comput 16:587–600. 10.1021/acs.jctc.9b00823 PMC7391796 31815476

[121] Britel O, Fitri A, Touimi Benjelloun A et al (2023) Carbazole based D-*π*i-*π*-A dyes for DSSC applications: DFT/TDDFT study of the influence of *π*i-spacers on the photovoltaic performance. Chem Phys 565:111738. 10.1016/j.chemphys.2022.111738

[122] Flores-Holguín N, Salas-Leiva JS, Glossman-Mitnik D (2023) Talarolide A and talaropeptides A–D: potential marine-derived therapeutic peptides with interesting chemistry and biological activity studied through density functional theory (DFT) and conceptual DFT. Molecules 28:6708. 10.3390/molecules28186708 PMC10536153 37764483

[123] Abdel Aal S, Awadh D (2023) The effect of central transition metals and electron-donating substituent on the performances of dye/TiO2 interface for dye-sensitized solar cells applications. J Mol Graph Model 123:108525. 10.1016/j.jmgm.2023.108525 37229869

[124] Geerlings P, De Proft F, Langenaeker W (2003) Conceptual density functional theory. Chem Rev 103:1793–1874. 10.1021/cr990029p 12744694

[125] Guido CA, Knecht S, Kongsted J, Mennucci B (2013) Benchmarking time-dependent density functional theory for excited state geometries of organic molecules in gas-phase and in solution. J Chem Theory Comput 9:2209–2220. 10.1021/ct400021c 26583715

