# Supplementary material for: (TD)DFT investigation of donor influence on the electronic and photovoltaic properties of D-π-A-π-A quinoxaline dyes
Source: J Mol Model. 2026 Sep 16;32(10):372. doi: 10.1007/s00894-026-06944-9 (PMC13582136; doi:10.1007/s00894-026-06944-9)
Supplement: Supplementary file 1 [file 894_2026_6944_MOESM1_ESM.docx]

Supporting Information

**(TD)DFT INVESTIGATION OF DONOR INFLUENCE ON THE ELECTRONIC AND PHOTOVOLTAIC PROPERTIES OF D-π-A-π-A QUINOXALINE DYES**

Malúcia M. Soeiro^1^, Nathália M.P. Rosa^2^, Itamar Borges Jr^1*^

*Instituto Militar de Engenharia, Departamento de Química. Praça Gen. Tibúrcio. 80 - Urca. Rio de Janeiro, Brazil - RJ. 22290-270.*

*^2^Instituto de Química. Universidade Federal Rural do Rio de Janeiro (UFRRJ). Seropédica. Rio de Janeiro - RJ. 23890-000. Brazil.*

*Corresponding author: [itamar@ime.eb.br](mailto:itamar@ime.eb.br)

**Table of Contents**

[**Section 1 - Supplementary data**	3](#_Toc239955091)

[**Table S1.** Frontier molecular orbital energy values HOMO and LUMO and gap ($Eg$) in eV using different DFT functionals in the gas phase.	3](#_Toc239955092)

[**Table S2.** TDDFT electronic properties of QX-DMA and QX-TPA sensitizers using different functionals in the gas phase. Compared with experimental wavelength (λ_max_) values (nm).	3](#_Toc239955093)

[**Table S3.** TDDFT electronic properties of QX-DMA and QX-TPA sensitizers using different functionals in THF solvent. Compared with experimental wavelength (λmax) values (nm).	4](#_Toc239955094)

[**Table S4**. Deviations between calculated and experimental HOMO and LUMO energies (eV) for the QX-DMA and QX-TPA dyes.	4](#_Toc239955095)

[**Table S5**. Comparative results obtained using the conventional B3LYP functional and the dispersion-corrected B3LYP-D3(BJ) approach: selected bond lengths $L$ (in Å) and dihedral angles $\Phi$ (in °) for the QX-DMA and QX-TPA dyes.	4](#_Toc239955096)

[**Table S6.** QX-DMA: excitation energies (E, eV), charge-transfer indices ($q(CT)$), participation numbers ($PRNTO$), hole-electron separations (and hole-electron separations $dex(Å)$ using the B3LYP/6-311G(d,p) level.	5](#_Toc239955097)

[**Table S7.** QX-TPA: excitation energies (E, eV), charge-transfer indices ($q(CT)$), participation numbers ($PRNTO$), hole-electron separations (and hole-electron separations $dex(Å)$.	5](#_Toc239955098)

[**Table S8.** CAM-B3LYP/6-311G(d,p) results for QX-DMA: oscillator strengths ($f$), excitation energies (eV), charge-transfer indices ($q\mathrm{CT}$ in $e$ units), participation numbers ($PRNTO$), and hole-electron separations $dex(Å)$ using the B3LYP/6-311G(d,p) level.	6](#_Toc239955099)

[**Table S9.** $\omega$B97X/6-311G(d,p) results for QX-DMA: oscillator strengths ($f$), excitation energies (eV), charge-transfer indices ($q\mathrm{CT}$ in *e* units), participation numbers ($PRNTO$), and hole-electron separations $dex(Å)$.	6](#_Toc239955100)

[**Table S10.** CAM-B3LYP/6-311G(d,p) results for QX-TPA: oscillator strengths ($f$), excitation energies (eV), charge-transfer indices ($q\mathrm{CT}$in *e* units), participation numbers ($PRNTO$), and hole-electron separations $dex(Å)$ using the B3LYP/6-311G(d,p) level.	6](#_Toc239955101)

[**Table S11.** $\omega$B97X /6-311G(d,p) results for QX-TPA: oscillator strengths ($f$), excitation energies (eV), charge-transfer indices ($q(CT)$ in *e* units), participation numbers ($PRNTO$) and hole-electron separations $dex(Å)$.	7](#_Toc239955102)

[**Fig S1** B3LYP/6-311G(d.p) optimized structures of the QX-DMA and QX-TPA dyes.	7](#_Toc239955103)

[**Fig S2.** QX-DMA two-dimensional representation of the $\Omega$ matrix, which describes the electron-hole separation. The figure also shows the dominant NTOs and the corresponding excitation energies for the higher-energy excited states $S2,S4 \mathrm{and} S5$.	8](#_Toc239955104)

[**Fig S3.** QX-DMA two-dimensional representation of the $\Omega$ matrix, which describes the electron-hole separation. The figure also shows the dominant NTOs and the corresponding excitation energies for the higher-energy excited states $S6,S7 ,S9 \mathrm{and} S10$.	9](#_Toc239955105)

[**Fig S4.** QX-TPA two-dimensional representation of the $\Omega$ matrix, which describes the electron-hole separation. The figure also shows the dominant NTOs and the corresponding excitation energies for the higher-energy excited states $S2, S4 \mathrm{and} S5$.	10](#_Toc239955106)

[**Fig S5.** QX-TPA two-dimensional representation of the $\Omega$ matrix, which describes the electron-hole separation. The figure also shows the dominant NTOs and the corresponding excitation energies for the higher-energy excited states $S6,S8 ,S9 \mathrm{and} S10$.	11](#_Toc239955107)

[**Fig S6** QX-DMA two-dimensional representation of the $\Omega$ matrix, which describes the electron-hole separation. The figure also shows the dominant NTOs and the corresponding excitation energies for the higher-energy excited states $S1-S10$. Calculated at the CAM-B3LYP/6-311G(d,p) level.	14](#_Toc239955108)

[**Fig S7** QX-TPA two-dimensional representation of the $\Omega$ matrix, which describes the electron-hole separation. The figure also shows the dominant NTOs and the corresponding excitation energies for the higher-energy excited states $S1$ - $S10$. Calculated at the CAM-B3LYP/6-311G(d,p) level.	17](#_Toc239955109)

[**Fig S8.** QX-DMA two-dimensional representation of the $\Omega$ matrix, which describes the electron-hole separation. The figure also shows the dominant NTOs and the corresponding excitation energies for the higher-energy excited states $S1.$- $S10$. Calculated at the $\omega$B97X/6-311G(d,p) level.	20](#_Toc239955110)

[**Fig S9.** QX-TPA two-dimensional representation of the $\Omega$ matrix, which describes the electron-hole separation. The figure also shows the dominant NTOs and the corresponding excitation energies for the higher-energy excited states $S1$ - $S10.$Calculated at the $\omega$B97X/6-311G(d,p) level.	23](#_Toc239955111)

[**Fig S10** Fragment-based analysis of the excited states $S1$ - $S10$. Contributions of charge transfer (CT) and Local Excitation (LE) between the fragments DMA (D), 2,3-diphenylquinoxaline $(A1)$, and cyanoacrylic acid $(A2)$. Calculated at the CAM-B3LYP/6-311G(d,p) level.	24](#_Toc239955112)

[**Fig S11** Fragment-based analysis of the excited states $S1$ - $S10$. Contributions of charge transfer (CT) and Local Excitation (LE) between the fragments TPA (D), 2,3-diphenylquinoxaline $(A1)$, and cyanoacrylic acid $(A2)$. Calculated at the CAM-B3LYP/6-311G(d,p) level.	24](#_Toc239955113)

[**Fig S12.** Fragment-based analysis of the excited states $S1$ - $S10$.Contributions of charge transfer (CT) and Local Excitation (LE) between the fragments DMA (D), 2,3-diphenylquinoxaline $(A1)$, and cyanoacrylic acid $(A2)$. Calculated at the $\omega$B97X/6-311G(d,p) level.	25](#_Toc239955114)

[**Fig S13**. Fragment-based analysis of the excited states $S1$ - $S10$. Contributions of charge transfer (CT) and Local Excitation (LE) between the fragments TPA (D), 2,3-diphenylquinoxaline $(A1)$, and cyanoacrylic acid $(A2)$. Calculated at the 𝜔B97X/6-311G(d,p) level.	25](#_Toc239955115)

[**Section 2 - Script for the Calculation of the Short-Circuit Current Density (**$\boldsymbol{JSC}$**)**	26](#_Toc239955116)

# **Section 1 - Supplementary data**

# **Table S1.** Frontier molecular orbital energy values HOMO and LUMO and gap ($E_{g}$) in eV using different DFT functionals in the gas phase.

| Dyes | Functional | $E_{HOMO}$ / eV | $E_{LUMO}$ / eV | $E_{g}$ |
| --- | --- | --- | --- | --- |
| QX-DMA | Expt. | -4.9 | -2.74 | 2.16 |
|  | B3LYP | -4.97 | -2.97 | 2.00 |
|  | CAM-B3LYP | -6.25 | -2.04 | 4.21 |
|  | wB97X | -7.09 | -1.27 | 5.82 |
|  | M06-2X | -6.20 | -2.35 | 3.85 |
|  | PBE | -4.56 | -3.41 | 1.14 |
| QX-TPA | Expt. | -5.18 | -3.09 | 2.09 |
|  | B3LYP | -5.06 | -3.01 | 2.05 |
|  | CAM-B3LYP | -6.34 | -2.14 | 4.20 |
|  | wB97X | -7.21 | -1.37 | 5.84 |
|  | M06-2X | -6.31 | -2.46 | 3.85 |
|  | PBE | -4.59 | -3.41 | 1.18 |

$$E_{g}= E_{LUMO}-E_{HOMO}$$

# **Table S2.** TDDFT electronic properties of QX-DMA and QX-TPA sensitizers using different functionals in the gas phase. Compared with experimental wavelength (λ_max_) values (nm).

| Dyes | Functional | λmax(calc.) | Δλ(shift) |
| --- | --- | --- | --- |
| QX-DMA | B3LYP | 627.0 | -95 |
|  | CAM-B3LYP | 469.8 | 62.2 |
|  | wB97X | 421.3 | 110.7 |
|  | M06-2X | 475.4 | 56 |
|  | PBE | 837.0 | -305 |
|  | Expt in THF ^a^ | 532 |  |
| QX-TPA | B3LYP | 645.2 | -132 |
|  | CAM-B3LYP | 462.1 | 59.9 |
|  | wB97X | 415.9 | 106.1 |
|  | M06-2X | 467.4 | 54.6 |
|  | PBE | 943.5 | -421.5 |
|  | Expt in THF^a^ | 522 |  |

^a^ Al-Marhabi AR. El-Shishtawy RM. Al-Footy KO. et al (2025) The impact of aryl amine donors on the performance of dye-sensitized solar cells based on quinoxaline D-π-A-π-A sensitizers. J Mol Struct 1333:141746. <https://doi.org/10.1016/j.molstruc.2025.141746>

# **Table S3.** TDDFT electronic properties of QX-DMA and QX-TPA sensitizers using different functionals in THF solvent. Compared with experimental wavelength (λmax) values (nm).

| Dyes | Functional | λ_max_(calc.) | Δλ(shift) |
| --- | --- | --- | --- |
| QX-DMA | B3LYP | 692.0 | -160 |
|  | CAM-B3LYP | 491.5 | 40.5 |
|  | wB97X | 436.9 | 95.1 |
|  | M06-2X | 499.8 | 32 |
|  | PBE | 983.1 | 56.6 |
|  | Expt. In THF | 532 |  |
| QX-TPA | B3LYP | 682.0 | -160 |
|  | CAM-B3LYP | 478.2 | 43.8 |
|  | wB97X | 429.4 | 92.6 |
|  | M06-2X | 484.0 | 38 |
|  | PBE | 1048.2 | -526.2 |
|  | Expt. in THF | 522 |  |

^a^ Al-Marhabi AR. El-Shishtawy RM. Al-Footy KO. et al (2025) The impact of aryl amine donors on the performance of dye-sensitized solar cells based on quinoxaline D-π-A-π-A sensitizers. J Mol Struct 1333:141746. <https://doi.org/10.1016/j.molstruc.2025.141746>

# **Table S4**. Deviations between calculated and experimental HOMO and LUMO energies (eV) for the QX-DMA and QX-TPA dyes.

| Dye | Functional | ${\Delta E}_{HOMO}$ | ${\Delta E}_{LUMO}$ |
| --- | --- | --- | --- |
| QX-DMA | B3LYP | 0.07 | 0.23 |
|  | CAM-B3LYP | 1.35 | 0.70 |
|  | wB97X | 2.19 | 1.47 |
|  | M06-2X | 1.30 | 0.39 |
|  | PBE | 0.34 | 0.67 |
| QX-TPA | B3LYP | 0.12 | 0.08 |
|  | CAM-B3LYP | 1.16 | 0.95 |
|  | wB97X | 2.03 | 1.72 |
|  | M06-2X | 1.13 | 0.63 |
|  | PBE | 0.59 | 0.32 |

$$\boldsymbol{\Delta}E = \left| E^{calc} -E^{expt} \right|$$

# **Table S5**. Comparative results obtained using the conventional B3LYP functional and the dispersion-corrected B3LYP-D3(BJ) approach: selected bond lengths $L$ (in Å) and dihedral angles $\Phi$ (in °) for the QX-DMA and QX-TPA dyes.

| Dyes | Functional | $L_{1}$ | $L_{2}$ | $L_{3}$ | $L_{4}$ | $\Phi_{1}$ | $\Phi_{2}$ | $\Phi_{3}$ | $\Phi_{4}$ |
| --- | --- | --- | --- | --- | --- | --- | --- | --- | --- |
| QX-DMA | B3LYP-D3 | 1.45 | 1.45 | 1.45 | 1.42 | -179.9 | 179.4 | 179.7 | -179.9 |
| QX-TPA | B3LYP-D3 | 1.45 | 1.45 | 1.45 | 1.42 | 179.9 | 179.2 | 179.7 | 179.9 |

**Table S6.** QX-DMA: excitation energies (E, eV), charge-transfer indices ($q(CT)$), participation numbers (${PR}_{NTO}$), hole-electron separations (and hole-electron separations $\tilde{d_{ex}}(Å)$ using the B3LYP/6-311G(d,p) level.

| States | E(eV) | $f$ | $\mathrm{PR}_{NTO}$ | $q(CT)$ | $\tilde{d_{ex}}(Å)$ |
| --- | --- | --- | --- | --- | --- |
| S_1_ | 1.790 | 1.08 | 1.019 | 0.767 | 10.515 |
| S_2_ | 2.389 | 0.02 | 1.136 | 0.729 | 10.891 |
| S_3_ | 2.646 | 0.77 | 1.178 | 0.673 | 8.295 |
| S_4_ | 2.992 | 0.04 | 2.01 | 0.492 | 7.601 |
| S_5_ | 3.071 | 0.21 | 2.159 | 0.606 | 8.177 |
| S_6_ | 3.176 | 0.09 | 1.465 | 0.743 | 9.800 |
| S_7_ | 3.232 | 0.028 | 1.027 | 0.310 | 5.139 |
| S_8_ | 3.323 | 0.73 | 1.919 | 0.577 | 7.686 |
| S_9_ | 3.486 | 0.0019 | 2.690 | 0.598 | 7.266 |
| S_10_ | 3.520 | 0.013 | 1.169 | 0.450 | 7.538 |

**Table S7.** QX-TPA: excitation energies (E, eV), charge-transfer indices ($q(CT)$), participation numbers ($PR_{NTO}$), hole-electron separations (and hole-electron separations $\tilde{d_{ex}}(Å)$.

| States | *f* | E(eV) | $PR_{NTO}$ | q(CT) | $\tilde{d_{ex}}(Å)$ |
| --- | --- | --- | --- | --- | --- |
| S_1_ | 0.94 | 1.818 | 1.016 | 0.805 | 11.700 |
| S_2_ | 0.34 | 2.442 | 1.985 | 0.657 | 11.840 |
| S_3_ | 0.53 | 2.536 | 2.000 | 0.810 | 9.739 |
| S_4_ | 0.11 | 2.977 | 1.255 | 0.515 | 7.959 |
| S_5_ | 0.18 | 3.055 | 2.099 | 0.705 | 9.969 |
| S_6_ | 0.14 | 3.114 | 1.772 | 0.640 | 9.385 |
| S_7_ | 0.67 | 3.203 | 2.200 | 0.532 | 7.968 |
| S_8_ | 0.004 | 3.222 | 1.015 | 0.298 | 5.022 |
| S_9_ | 0.027 | 3.376 | 2.569 | 0.663 | 8.160 |
| S_10_ | 0.008 | 3.538 | 1.050 | 0.427 | 7.653 |

# **Table S8.** CAM-B3LYP/6-311G(d,p) results for QX-DMA: oscillator strengths ($f$), excitation energies (eV), charge-transfer indices ($q\left( \mathrm{CT} \right)$ in $e$ units), participation numbers ($PR_{NTO}$), and hole-electron separations $\tilde{d_{ex}}(Å)$ using the B3LYP/6-311G(d,p) level.

| States | *f* | E(eV) | $PR_{NTO}$ | $q(CT)$ | $\tilde{d_{ex}}(Å)$ |
| --- | --- | --- | --- | --- | --- |
| S_1_ | 1.78 | 2.52 | 1.7 | 0.90 | 12.7 |
| S_2_ | 0.27 | 3.36 | 2.5 | 0.68 | 8.15 |
| S_3_ | 0.03 | 3.60 | 2.3 | 0.69 | 10.0 |
| S_4_ | 0.014 | 3.74 | 1.84 | 0.60 | 7.27 |
| S_5_ | 0.16 | 3.76 | 1.74 | 0.75 | 7.94 |
| S_6_ | 0.64 | 4.0 | 1.78 | 0.62 | 8.61 |
| S_7_ | 0.50 | 4.2 | 2.2 | 0.76 | 8.82 |
| S_8_ | 0.052 | 4.3 | 2.4 | 0.70 | 8.0 |
| S_9_ | 0.13 | 4.8 | 2.3 | 0.55 | 7.60 |
| S_10_ | 0.18 | 4.5 | 2.1 | 0.75 | 8.40 |

# **Table S9.** $\omega$B97X/6-311G(d,p) results for QX-DMA: oscillator strengths ($f$), excitation energies (eV), charge-transfer indices ($q\left( \mathrm{CT} \right)$ in *e* units), participation numbers ($PR_{NTO}$), and hole-electron separations $\tilde{d_{ex}}(Å)$.

| States | *f* | E(eV) | $PR_{NTO}$ | $q(CT)$ | $\tilde{d_{ex}}(Å)$ |
| --- | --- | --- | --- | --- | --- |
| $S_{1}$ | 1.97 | 2.84 | 2.123 | 0.361 | 5.401 |
| $S_{2}$ | 0.12 | 3.63 | 2.412 | 0.26 | 4.669 |
| $S_{3}$ | 0.015 | 3.87 | 1.1 | 0.079 | 3.186 |
| $S_{4}$ | 0.215 | 4.03 | 1.271 | 0.138 | 4.296 |
| $S_{5}$ | 0.471 | 4.22 | 2.057 | 0.377 | 5.526 |
| $S_{6}$ | 0.274 | 4.62 | 2.39 | 0.564 | 7.942 |
| $S_{7}$ | 0.1659 | 4.63 | 2.101 | 0.188 | 4.258 |
| $S_{8}$ | 0.0367 | 4.68 | 1.258 | 0.168 | 3.442 |
| $S_{9}$ | 0.553 | 4.75 | 2.275 | 0.235 | 4.512 |
| $S_{10}$ | 0.127 | 4.91 | 2.253 | 0.321 | 5.332 |

# **Table S10.** CAM-B3LYP/6-311G(d,p) results for QX-TPA: oscillator strengths ($f$), excitation energies (eV), charge-transfer indices ($q\left( \mathrm{CT} \right)$in *e* units), participation numbers ($PR_{NTO}$), and hole-electron separations $\tilde{d_{ex}}(Å)$ using the B3LYP/6-311G(d,p) level.

| States | *f* | E(eV) | $PR_{NTO}$ | $q(CT)$ | $\tilde{d_{ex}}(Å)$ |
| --- | --- | --- | --- | --- | --- |
| S_1_ | 1.94 | 2.6 | 1.143 | 0.44 | 6.579 |
| S_2_ | 0.12 | 3.4 | 1.893 | 0.438 | 6.642 |
| S_3_ | 0.13 | 3.6 | 1.93 | 0.519 | 8.862 |
| S_4_ | 0.034 | 3.74 | 1.15 | 0.172 | 4.896 |
| S_5_ | 0.182 | 3.75 | 1.67 | 0.225 | 5.258 |
| S_6_ | 0.764 | 3.94 | 1.32 | 0.442 | 7.669 |
| S_7_ | 0.155 | 4.1 | 2.1 | 0.542 | 7.60 |
| S_8_ | 0.047 | 4.3 | 1.3 | 0.120 | 4.19 |
| S_9_ | 0.094 | 4.3 | 2.78 | 0.344 | 6.11 |
| S_10_ | 0.21 | 4.4 | 2.31 | 0.406 | 7.70 |

# **Table S11.** $\omega$B97X /6-311G(d,p) results for QX-TPA: oscillator strengths ($f$), excitation energies (eV), charge-transfer indices ($q(CT)$ in *e* units), participation numbers ($PR_{NTO}$) and hole-electron separations $\tilde{d_{ex}}(Å)$.

| States | *f* | E(eV) | $PR_{NTO}$ | $q(CT)$ | $\tilde{d_{ex}}(Å)$ |
| --- | --- | --- | --- | --- | --- |
| $S_{1}$ | 2.087 | 2.90 | 1.2 | 0.348 | 5.255 |
| $S_{2}$ | 0.056 | 3.65 | 2.4 | 0.245 | 4.652 |
| $S_{3}$ | 0.025 | 3.86 | 1.1 | 0.079 | 3.191 |
| $S_{4}$ | 0.0247 | 4.04 | 1.8 | 0.137 | 4.288 |
| $S_{5}$ | 0.696 | 4.16 | 2.4 | 0.259 | 4.882 |
| $S_{6}$ | 0.041 | 4.56 | 1.7 | 0.175 | 4.557 |
| $S_{7}$ | 0.138 | 4.60 | 2.7 | 0.354 | 6.056 |
| $S_{8}$ | 0.0820 | 4.70 | 2.2 | 0.325 | 6.253 |
| $S_{9}$ | 0.340 | 4.72 | 2.7 | 0.285 | 5.785 |
| $S_{10}$ | 0.382 | 4.76 | 1.1 | 0.027 | 3.6 |

# **Fig S1** B3LYP/6-311G(d.p) optimized structures of the QX-DMA and QX-TPA dyes.

# **Fig S2.** QX-DMA two-dimensional representation of the $\Omega$ matrix, which describes the electron-hole separation. The figure also shows the dominant NTOs and the corresponding excitation energies for the higher-energy excited states $S_{2}{,S}_{4}\mathrm{and} S_{5}$.

# **Fig S3.** QX-DMA two-dimensional representation of the $\Omega$ matrix, which describes the electron-hole separation. The figure also shows the dominant NTOs and the corresponding excitation energies for the higher-energy excited states $S_{6}{,S}_{7},S_{9}\mathrm{and} S_{10}$.

# **Fig S4.** QX-TPA two-dimensional representation of the $\Omega$ matrix, which describes the electron-hole separation. The figure also shows the dominant NTOs and the corresponding excitation energies for the higher-energy excited states $S_{2}{, S}_{4}\mathrm{and} S_{5}$.

# **Fig S5.** QX-TPA two-dimensional representation of the $\Omega$ matrix, which describes the electron-hole separation. The figure also shows the dominant NTOs and the corresponding excitation energies for the higher-energy excited states $S_{6}{,S}_{8},S_{9}\mathrm{and} S_{10}$.

**Fig S6** QX-DMA two-dimensional representation of the $\Omega$ matrix, which describes the electron-hole separation. The figure also shows the dominant NTOs and the corresponding excitation energies for the higher-energy excited states $S_{1}-S_{10}$. Calculated at the CAM-B3LYP/6-311G(d,p) level.

# **Fig S7** QX-TPA two-dimensional representation of the $\Omega$ matrix, which describes the electron-hole separation. The figure also shows the dominant NTOs and the corresponding excitation energies for the higher-energy excited states $S_{1}$ - $S_{10}$. Calculated at the CAM-B3LYP/6-311G(d,p) level.

# **Fig S8.** QX-DMA two-dimensional representation of the $\Omega$ matrix, which describes the electron-hole separation. The figure also shows the dominant NTOs and the corresponding excitation energies for the higher-energy excited states $S_{1}.$- $S_{10}$. Calculated at the $\omega$B97X/6-311G(d,p) level.

# **Fig S9.** QX-TPA two-dimensional representation of the $\Omega$ matrix, which describes the electron-hole separation. The figure also shows the dominant NTOs and the corresponding excitation energies for the higher-energy excited states $S_{1}$ - $S_{10}.$Calculated at the $\omega$B97X/6-311G(d,p) level.

# **Fig S10** Fragment-based analysis of the excited states $S_{1}$ - $S_{10}$. Contributions of charge transfer (CT) and Local Excitation (LE) between the fragments DMA (D), 2,3-diphenylquinoxaline $(A1)$, and cyanoacrylic acid $(A2)$. Calculated at the CAM-B3LYP/6-311G(d,p) level.

# **Fig S11** Fragment-based analysis of the excited states $S_{1}$ - $S_{10}$. Contributions of charge transfer (CT) and Local Excitation (LE) between the fragments TPA (D), 2,3-diphenylquinoxaline $(A1)$, and cyanoacrylic acid $(A2)$. Calculated at the CAM-B3LYP/6-311G(d,p) level.

# **Fig S12.** Fragment-based analysis of the excited states $S_{1}$ - $S_{10}$.Contributions of charge transfer (CT) and Local Excitation (LE) between the fragments DMA (D), 2,3-diphenylquinoxaline $(A1)$, and cyanoacrylic acid $(A2)$. Calculated at the $\omega$B97X/6-311G(d,p) level.

# **Fig S13**. Fragment-based analysis of the excited states $S_{1}$ - $S_{10}$. Contributions of charge transfer (CT) and Local Excitation (LE) between the fragments TPA (D), 2,3-diphenylquinoxaline $(A1)$, and cyanoacrylic acid $(A2)$. Calculated at the 𝜔B97X/6-311G(d,p) level.

# **Section 2 - Script for the Calculation of the Short-Circuit Current Density (**$\boldsymbol{J}_{\boldsymbol{SC}}$**)**

from google.colab import files

uploaded = files.upload('AM15.xlsx') # Select the AM15.xlsx file from your PC.

import pandas as pd

import numpy as np

from scipy.integrate import simpson

from google.colab import files

\# === Read the AM1.5G file ===

df_am15 = pd.read_excel('AM15.xlsx/AM15.xlsx')

lambda_am1 = df_am15['Wvlgth nm'].values

phi_am1 = df_am15['phi_G m-2s-1'].values # Choose any flux column you want.

\# === your data ===

lambda_nm = np.array([350.4, 364.2, 384.8, 387.0, 398.1, 405.8, 416.4, 488.9, 507.7, 682.0])

f_osc = np.array([0.01, 0.03, 0.01, 0.67, 0.15, 0.19, 0.12, 0.53, 0.35, 0.94])

phi_injection = 7.24

eta_collection = 1.0

eta_regeneration = 1.0

\# Interpolate the solar irradiance to the wavelengths of your data.

phi_solar = np.interp(lambda_nm, lambda_am1, phi_am1)

def calcular_Jsc(lambda_nm, f_osc, phi_injection, phi_solar, eta_collection=1.0, q=1.602176634e-19):

lambda_nm = np.array(lambda_nm)

if np.isscalar(f_osc):

f_osc = np.full_like(lambda_nm, f_osc, dtype=float)

else:

f_osc = np.array(f_osc, dtype=float)

if np.isscalar(phi_injection):

phi_injection = np.full_like(lambda_nm, phi_injection, dtype=float)

else:

phi_injection = np.array(phi_injection, dtype=float)

phi_solar = np.array(phi_solar, dtype=float)

LHE = 1 - 10**(-f_osc)

integrando = phi_solar * LHE * phi_injection * eta_collection

integral = simpson(integrando, x=lambda_nm)

return integral

Jsc = calcular_Jsc(lambda_nm, f_osc, phi_injection, phi_solar, eta_collection)

print("J_SC =", Jsc, "A/m²")

print("J_SC =", Jsc*1e3/1e4, "mA/cm²")
